# *De novo* draft assembly of the *Botrylloides leachii* genome provides further insight into tunicate evolution

**DOI:** 10.1038/s41598-018-23749-w

**Published:** 2018-04-03

**Authors:** Simon Blanchoud, Kim Rutherford, Lisa Zondag, Neil J. Gemmell, Megan J. Wilson

**Affiliations:** 1 Department of Anatomy, School of Biomedical Sciences, University of Otago, P.O. Box 56, Dunedin, 9054 New Zealand; 2 Present Address: Department of Biology, University of Fribourg, Fribourg, Switzerland

## Abstract

Tunicates are marine invertebrates that compose the closest phylogenetic group to the vertebrates. These chordates present a particularly diverse range of regenerative abilities and life-history strategies. Consequently, tunicates provide an extraordinary perspective into the emergence and diversity of these traits. Here we describe the genome sequencing, annotation and analysis of the Stolidobranchian *Botrylloides leachii*. We have produced a high-quality 159 Mb assembly, 82% of the predicted 194 Mb genome. Analysing genome size, gene number, repetitive elements, orthologs clustering and gene ontology terms show that *B. leachii* has a genomic architecture similar to that of most solitary tunicates, while other recently sequenced colonial ascidians have undergone genome expansion. In addition, ortholog clustering has identified groups of candidate genes for the study of colonialism and whole-body regeneration. By analysing the structure and composition of conserved gene linkages, we observed examples of cluster breaks and gene dispersions, suggesting that several lineage-specific genome rearrangements occurred during tunicate evolution. We also found lineage-specific gene gain and loss within conserved cell-signalling pathways. Such examples of genetic changes within conserved cell-signalling pathways commonly associated with regeneration and development that may underlie some of the diverse regenerative abilities observed in tunicates. Overall, these results provide a novel resource for the study of tunicates and of colonial ascidians.

## Introduction

**Figure 1:**
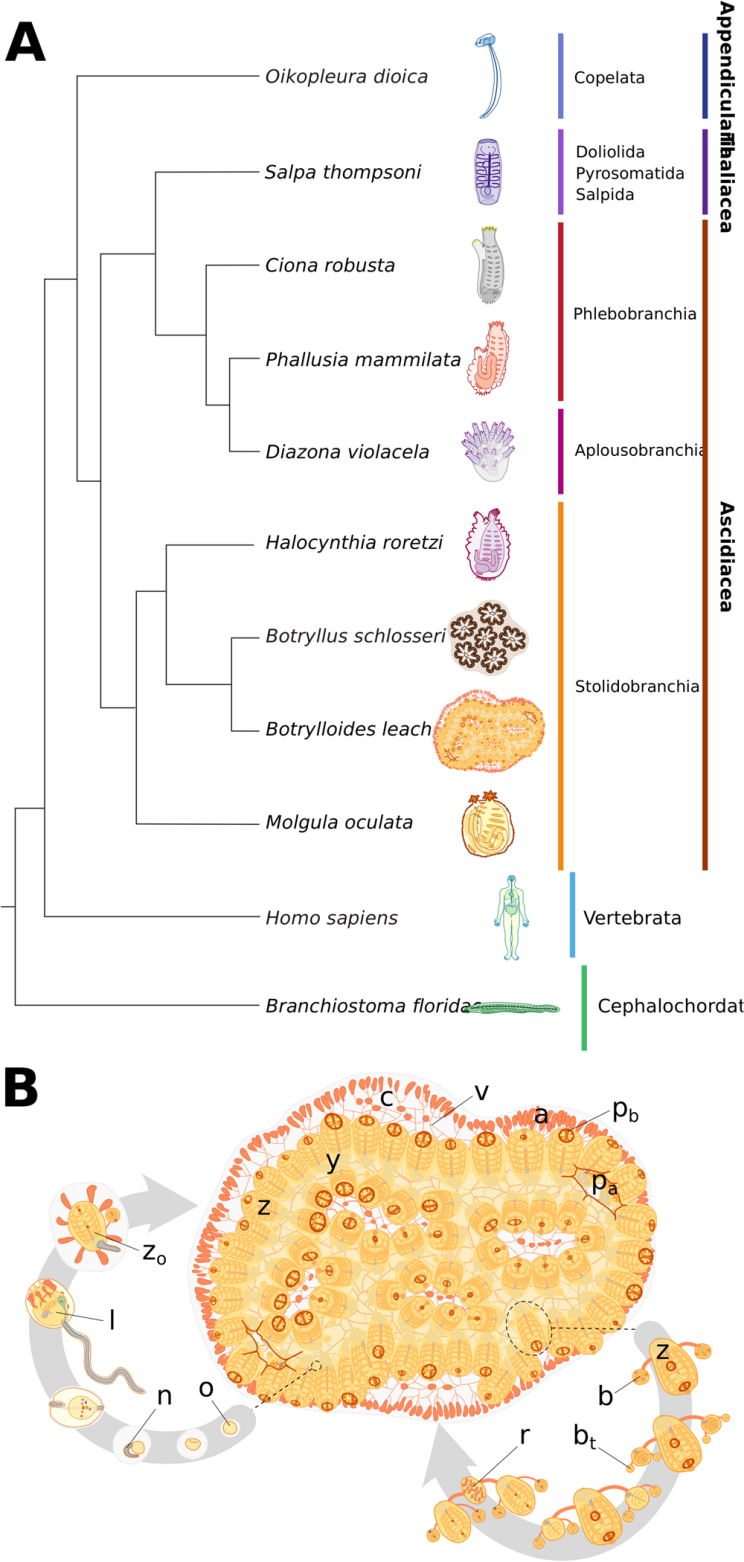
*B. leachii* phylogenetic position and life cycle. (**A**) Schematic showing phylogeny of tunicates with respect to the chordate clade (consensus based on^4,146,147^). (**B**) Life cycle of *B. leachii*. The colony expands and grows by asexual reproduction (right loop). During favourable conditions such as warmer water temperatures, members of the colonies start sexual reproduction (left loop). The embryo develops viviparously within the colony in brood pouches until hatching. Motile larvae attach to nearby substrates and begin metamorphosis into oozooids. Abbreviations: zooid (z), system (y), tunic (c), vascular system (v), terminal ampullae (a), buccal siphon (p_b_), atrial siphon (p_a_), fertilized oocyte (o), notochord (n), larval tadpole (l), oozooid (z_o_), bud (b), budlet (b_t_), regressing zooid (r).

Tunicates are a group of worldwide marine invertebrates, the majority of which are subtidal suspension-feeding hermaphrodites. This subphylum is part of the Chordata phylum, phylogenetically positioned between the more basal Cephalochordata and the higher Vertebrata, of which they are considered the closest relatives^1^ (Fig. 1A). These organisms include a wide range of reproductive methods, regenerative abilities, developmental strategies and life cycles^2^. Importantly, and despite a drastically different body plan during their adult life cycle, tunicates have a tissue complexity related to that of vertebrates (Fig. 1A), including a heart, a notochord, an endostyle and a vascular system^3^. In addition, this group of animals is undergoing rapid genomic evolution, with a greater nucleotide substitution rate observed in both their nuclear and mitochondrial genomes, when compared to vertebrates^4–7^. Therefore, this chordate subphylum provides an excellent opportunity to study the origin of vertebrates, the emergence of clade specific traits and the function of conserved molecular mechanisms. Biological features that can be investigated in tunicates include, among others, the evolution of colonialism, sessileness, and budding. Moreover, some compound tunicates can undergo whole-body regeneration (WBR), whereby a fully functional adult can be restored from a portion of vascular tissue^8^. The presence of such an extensive regenerative capacity, in the closest relatives of the vertebrates, renders the study of tunicates particularly well suited for comparative research. In particular, identifying and investigating the shared regulatory mechanisms and signalling pathways required for successful regeneration is of interest to regenerative medicine and ageing research^9–12^. However, there are currently only eight Tunicata genomes publicly available^7,13–15^, of which four have been well annotated.

Tunicates are separated into seven orders contained in three classes (Fig. 1A): Appendicularia (order Copelata), Thaliacea (orders Pyrosomida, Salpida and Doliolida) and Ascidiacea (orders Aplousobranchia, Phlebobranchia and Stolidobranchia). Appendicularia is a class of planktonic free-swimming organisms that possess chordate traits common to most tunicate larvae including a notochord, neural tube and pharyngeal slits. These social animals form communities where each individual is enclosed inside a special external mucous structure, termed house, which concentrates and funnels their food. *Oikopleura dioica* is the sole example of the Appendicularian to have its genome sequenced, showing exceptional compaction (70 Mb)^13^.

Thaliacea is a class of planktonic pelagic animals forming free-floating compound colonies^16^. These organisms can reproduce both sexually to initiate novel colonies, as well as asexually, through stolonial budding, to increase the size of the colony. Owing to their peculiar life cycle and habitat, these tunicates have been studied less thoroughly in comparison to other ascidians, and whether they can undergo regeneration remains unknown. A single species, *Salpa thompsoni*, has been sequenced^7^ and has a large and repetitive genome (602 Mb, 33% is repetitive sequences).

Ascidiacea consists of both solitary and colonial sessile benthic organisms. Solitary ascidians (Phlebobranchian and some families among the Stolidobranchian) reproduce sexually, releasing eggs through their atrial siphon for external fertilization, hence producing a motile larva. These larvae will explore their environment, attach to a submersed substrate and undergo metamorphosis into a sessile filter-feeding adult. These ascidians can regenerate some organs, including their oral siphon^17,18^ although regeneration capability reduces as they age^19^. Ascidiacean genomes represent the majority of the sequenced tunicate genomes, with five published genomes (*Ciona robusta* [formerly known as *C. intestinalis* type A], *Ciona savigny, Molgula oculata*, *Molgula occulta*, *Molgula occidentalis*^14,20–23^), two yet unpublished species (*Phallusia mamilata*, *Phallusia fumigata*^22^) and two currently being assembled (*Halocynthia rorezi*, *Halocynthia aurantium*^22^). These published genomes are estimated to be between 160–200 Mb (Table 1).

**Table 1:** Comparison of the sequenced tunicate genomes and their most prominent biological features.

	*Botryllus schlosseri*	*Ciona robusta*	*Ciona savignyi*	*Molgula occidentalis*	*Molgula occulta*	*Molgula oculata*	*Oikopleura dioica*	*Salpa thompsoni*	*Botrylloides leachii*
**Genome size**	725 Mb	160 Mb	190 Mb	160 Mb	160 Mb	160 Mb	72 Mb	602 Mb	194 Mb
**Number of scaffolds**	121,094	4,390	374	21,251	23,663	10,554	1,260	478,281	1,778
**Fraction of repetitive DNA**	60%	26%	35%	27%	23%	26%	15%	60–70%	19%
**Predicted gene number**	27,463	16,671	11,956	30,639	N/A	15,313	16,749	26,415	15839
**GC content**	41%	36%	37%	33%	38%	36%	40%	37%	41%
**Body structure**	colony, sessile	solitary, sessile	solitary, sessile	solitary, sessile	solitary, sessile	solitary, sessile	solitary. motile	colony, planktonic	colony, sessile
**Reproduction**	asexual, sexual, hermaphrodite	sexual, hermaphrodite	sexual, hermaphrodite	sexual, hermaphrodite	sexual, hermaphrodite	sexual, hermaphrodite	sexual, separated sexes	asexual, sexual, hermaphrodite	asexual, sexual, hermaphrodite
**Regenerative ability**	WBR	specific organs	specific organs	unknown	unknown	unknown	unknown	unknown	WBR

Colonial sessile tunicates (species found in the Aplousobranchia and Stolidobranchia orders) are capable of both sexual and asexual reproduction, through a wide range of budding types (palleal, vascular, stolonial, pyloric and strobilation^24^), as well as WBR. Colonial ascidians are emerging as increasingly popular model organisms for a variety of studies including immunobiology, allorecognition, angiogenesis and WBR^25–32^. Only a single colonial Stolidobranchia genome, *Botryllus schlosseri*, is publicly available, which revealed a considerable expansion of genome size (725 Mb, almost three fold) when compared to the other published ascidian genomes^15^. A second partially assembled, yet unpublished, genome of colonial ascidian appears to reflect a similar genome expansion (*Didemnum vexillum*, >542 Mb^33^). To provide a resource for further studies on the genetics and evolution of this subphylum, as well as research on colonialism and WBR, we have assembled and analysed the genome sequence of *Botrylloides leachii* (class Ascidiacea, order Stolidobranchia^34^).

The viviparous colonial ascidian *B. leachii* (Fig. 1B) lives in colonies composed of genetically identical adults (termed zooids) organized in ladder-like systems and embedded in gelatinous matrix (tunic). While each adult has its own heart, they all share a common vascular system embedded within the tunic. In the presence of sufficient food supply, the size of the colony doubles weekly through synchronized asexual reproduction, known as palleal budding^35^. During this process, each adult produces two daughter zooids that ultimately replace the mother, which is then resorbed by the colony (Fig. 1B). *B. leachii* can also reproduce sexually through a tadpole stage that allows the settlement of a new colony onto a substrate (Fig. 1B). Following removal or loss of all zooids from the colony, *B. leachii* can undergo WBR and restore a single fully-functional adult in as little as 10 days from a small piece of its vascular system^26^. Furthermore, when facing unfavourable environmental conditions, these colonial tunicates can enter into hibernation, whereby all zooids undergo regression and are resorbed by the remaining vascular system. When a favourable environment is restored, mature adults will develop to re-establish the colony^36^.

We have assembled and annotated the first *de novo* draft genome of *B. leachii* by taking advantage of our recently published transcriptomes^37^. Using this genome, we have then undertaken a large-scale comparison of the four best-annotated tunicate genomes *(B. schlosseri*, *C. robusta, M. oculat*a and *O. dioica*) to gain insights into some of the diverse biological abilities that have evolved within the Tunicata.

## Results

### Genome assembly and annotation

To minimize contamination from marine algae and bacteria typically present in the pharyngeal basket of feeding *B. leachii*, we isolated genomic DNA from embryos of a single wild *B. leachii* colony. Genomic DNA was used to produce two libraries: one short-range consisting of 19,090,212 fragments (300 bp) of which 100 bp were paired-end sequenced, important to obtain high coverage, and a second long-range mate pair with 31,780,788 fragments (1.5–15 kb size range, median ~3 kb) of which 250 bp were paired-end sequenced, to aid scaffold assembly. Following quality checks, low quality reads were removed and sequencing adaptors were trimmed, thus resulting in a high-quality dataset of 86,644,308 paired-end and 12,112,004 single-end sequences (100% with a mean Phred score >=30, <1% with an adapter sequence, Fig. S1A).

We then followed a reference-free genome characterization^38^ to estimate three properties of the *B. leachii* genome; provided with statistics from the human, fish (*Maylandia zebra*^39^), bird (*Melopsittacus undulatus*^39^) and oyster (*Crassostrea gigas*^40^) genomes for comparison. Firstly, the SGA-PreQC package^38^ was used to estimate the genome size to be 194 Mb (194,153,277 bp). This size is similar to that of the solitary *C. robusta, C. savigny and M. occidentalis, M. oculata* (160 Mb, 190 Mb, 160 Mb and 160 Mb, respectively^14,20,23^), larger than the compacted 70 Mb genome of *O. dioica*^13^ but appreciably smaller than the predicted 725 Mb genome of the closely related colonial ascidian *B. schlosseri*, of which 580 Mb have been sequenced^15^. Secondly, by quantifying the structure of the de Brujin graph obtained using the k-mer counts (k = 31), the computational complexity of the assembly was estimated (sequencing errors 1/213; allelic differences 1/233; genomic repeats 1/2,439). With a cumulative occurrence of 1/106, the *B. leachii* genome is similar to that of bird, more variable than those of fish and human, but still quite less complex than the notably difficult oyster genome^38^ (Fig. S1B). Lastly, sequence coverage was estimated using the distribution of 51-mers counts, showing a well-separated bimodal distribution with a true-genomic k-mers maximum at 31 × coverage, similar to the human genome but higher than both the fish and the bird. Overall, these metrics suggest that the raw sequencing data was suitable for *de novo* assembly.

*De novo* assembly using Metassembler^41^ produced a genome of 159,132,706 bp (estimated percentage of genome assembled is 82%), with an average sequencing coverage of 66x (after adaptor trimming). The assembly is composed of 1,778 scaffolds, with a N50 scaffold length of 209,776 and a L50 scaffold count of 223. The 7,783 contigs, with a N50 length of 48,085, and a L50 count of 781, represent a total size of 146,061,259 (92%, Table 2). To evaluate the completeness of our assembly, we used the Benchmarking Universal Single-Copy Orthologs (BUSCO^42^). This tool provides a quantitative measure of genome completeness by verifying the presence of a set of manually curated and highly conserved genes. Out of the 978 orthologs selected in metazoans, 866 (89%) were found in our assembly of the *B. leachii* genome (File S1), a relatively high score when compared to the BUSCO score of frequently used genome assemblies such as *Homo sapiens* (89%, GCA_000001405.15^42^). In addition, we took advantage of our previous assembly of the *B. leachii* transcriptome^37^ to further assess the quality of our genome. Using BLAT^43^, we were able to map 93% of transcript sequences (48,510/52,004) onto our assembly. Overall, these results indicate that the *B. leachii de novo* genome assembly was largely complete and suitable for annotation.

**Table 2:** *B. leachii* genome assembly statistics.

Total length of assembly	159,132,706 bp
Predicted genome size	194 Mb
Number of scaffolds	1,778
Median scaffold length	209,776 bp
N50 contig length	43,485 bp
Estimated genome coverage before adaptor trimming	101x
Estimated genome coverage after adaptor trimming	66x
Number of predicted genes	15,839
% of the *B. leachii* reference transcriptome aligning to the genome	93%
% of *Ciona* proteins that have a significant match to the *B. leachii* genome	71%
BUSCO score BUSCO notation assessment results	89% (866/978) C:89% [D:7.1%], F:4.0%, M:7.5%

*Ab initio* genome annotation was performed using MAKER2^44^ and predicted 15,839 coding genes, of which 13,507 could be classified using InterProScan^45^. Comparing these predictions with our mapping of the transcriptome, we found out that 83% of our aligned cDNA (40,188/48,510) mapped to a predicted gene locus thus spanning 78% of the annotated genes (12,395/15,839). In addition, a total of 4,213 non-coding sequences were predicted using Infernal^46^, Snoscan^47^ and tRNAscan-SE^48^. Finally, repetitive elements were annotated using RepeatMasker^49^ and a species-specific library created using the RepeatModeler module^50^. Ninteen percent of the genome was identified as containing repetitive elements (Table 2, File S2), a majority (17% out of 19%) of these being interspersed repeats.

To further characterize the genome of *B. leachii*, we compared it to four available Tunicata genomes. The proportion of repetitive elements in *B. leachii* is similar to other tunicates (File S2) including *C. robusta* (25%), *M. oculata* (24%) and *O. dioica* (15%), while being much lower than *B. schlosseri* (60%). In particular, there are at least two additional families in the *B. schlosseri* hAT transposon superfamily and counts for some hAT elements differ dramatically (e.g. hAT-Charlie 366 in *B. leachii* vs 46,661 in *B. schlosseri*; File S2). We then quantified the number of sequences from each proteome that mapped onto our assembly using tBLASTn^51^: *C. robusta* 71% (10,507/14,740), *M. oculata* 77% (12,788/16,616), *B. schlosseri* 71% (32,944/46,519) but only 30% for *O. dioica* (9,009/29,572).

**Figure 2:**
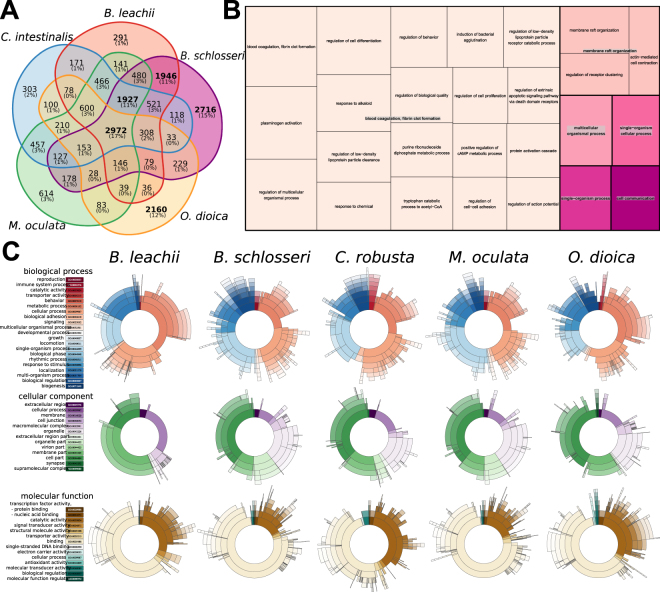
Comparison of tunicate genomes. (**A**) Clustering of orthologous protein sequences. Indicated are the number of cluster groups, each of which contains at least two proteins. (**B**) TreeMap representation of the overrepresented GO Biological Processes terms within the ortholog groups shared between *B. leachii* and *B. schlosseri* genomes but not with *C. robusta*, *O. dioica* and *M. oculata*. Each rectangle size is proportional to the GOrilla minimum hypergeometric *p*-value of each GO term. (**C**) Distribution of the three classes of GO terms for each species. The colour-codes (left) are common for the entire row.

Next, we performed an all-to-all search for protein orthologs between the tunicate genomes using the OrthoMCL clustering approach^52^ (Fig. 2A). Clustering the combined protein set from all five genomes resulted in 17,710 orthologous groups of annotated genes. By classifying each group based on which tunicate genome(s) they were present within, we identified five orthologous sets of genes: those shared by all species (17% of all groups, 2,972 groups), those shared by all sessile tunicates (11%, 1,927), those between two colonial species (11%, 1,946) and two groups unique to *B. schlosseri* and *O. dioica* (15% and 12%, 2,716 and 2,160, respectively; Fig. 2A). Lastly, these proteins specific to a single genome were removed from the corresponding proteome, and a new mapping onto our assembly was performed. Mapping of these two filtered proteomes reached 93% for *B. schlosseri* and 45% for *O. dioica*. Altogether, these results indicate that our *de novo* assembly is highly compatible with that of other tunicates and thus amenable for comparisons with their genomes.

To gain insights into the potential biological function underlying these ortholog groups, we analysed the distribution of Gene Ontology (GO) terms for each cluster and visualized these using REVIGO/Treemap (Figs 2B and S2). Given that the proteins identified by OrthoMCL clustering are potentially novel to colonial ascidians, a cross-species approach for GO enrichment was performed using the Human GO database as background^53^ (Fig. 2B). The overrepresented genes (Fig. 2B, File S4) function in biological processes such as circulation (GO:0003018, GO:0003013, GO:0050880), wound healing (GO:0072378) and cell communication (GO:0007154); as well as the regulation of immune cell differentiation (GO:0033081, GO:0033089) and immune system processes (GO:0002376, GO:0032608). These biological functions are concordant with that of proteins predicted to be required for the life cycle of colonial ascidian.

Finally, we compared the overall composition of GO terms for all five tunicates (Fig. 2C). Despite *B. schlosseri* having a larger predicted gene number compared to the other analysed tunicates, the overall proportion of GO group terms were distributed similarly between all genomes (Fig. 2C), indicating no expansion of one particular functional group in *B. schlosseri*.

Overall, the above analyses showed that our assembly and annotation are consistent with the other tunicate genomes and will provide additional insights into the Tunicata subphylum.

### Ancient gene linkages are fragmented in tunicate genomes

To gain further insights in the evolution of the Tunicate genomes, we investigated the organisation of three ancient gene clusters, representing highly conserved sets of genes that are typically located adjacent to each other within a genome^54^. These clusters arose in a common ancestor and were preserved because of shared regulatory mechanisms. The homeobox-containing *Hox* gene family^55^, typically composed of 13 members in vertebrates^56^, is among the best-studied examples of such an ancient gene cluster, and is critical for embryonic development^57^. The linear genomic arrangement of genes within the *Hox* cluster reflects their spatial expression along the anterior-posterior body axis, which establishes regional identity across this axis^57^.

**Figure 3:**
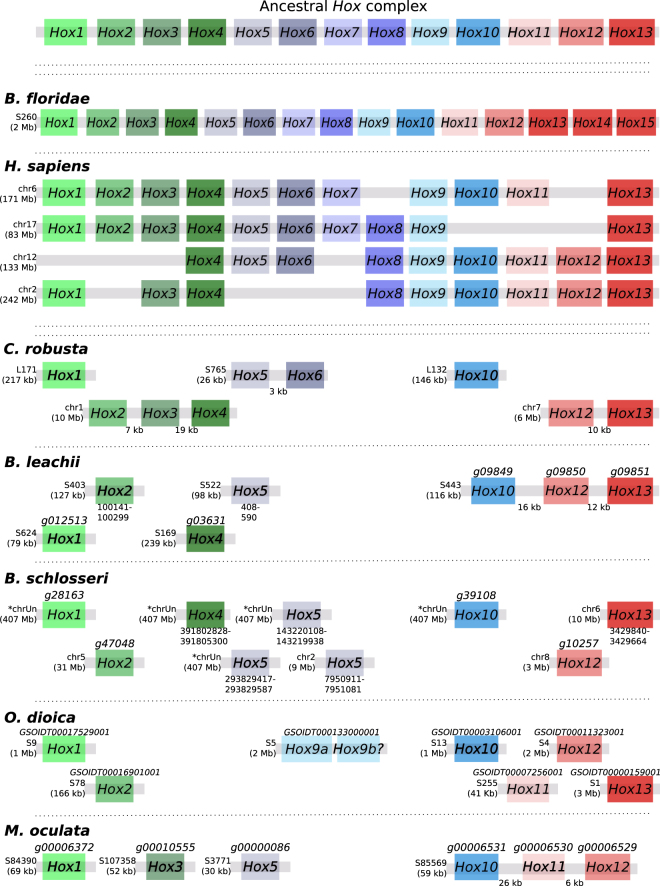
*Hox* genes are dispersed and reduced in number within tunicate genomes. Schematic depicting *Hox* gene linkages retained in five tunicate genomes in comparison to the ancestral *Hox* complex, which included thirteen genes. Orthologous genes are indicated by common colours. Chromosome (chr) or scaffold number (S) is shown, along with gene ID when available for newly annotated genomes. For *B. floridae* and *H. sapiens*, the length of each *Hox* gene cluster is given in brackets, and for *B. leachii*, the total scaffold length is shown. If a gene ID is not available (for unannotated genes), the co-ordinates of the BLAST hit (for the homeobox protein domain) is either given in File S5 or shown in the figure under the putative gene. Transcript IDs for *B. leachii Hox* genes identified in our transcriptome data^37^ are also provided in File S5.

The basal cephalochordate *Branchiostoma floridae* genome has all 13 *Hox* genes located in a single cluster, along with two additional *Hox* genes (Fig. 3), suggesting that the chordate ancestor also had an intact cluster^58^. However, in tunicates, this clustering appears to be lost^59–62^ (Fig. 3). In *C. robusta*, the nine identified *Hox* genes are distributed across five scaffolds, with linkages preserved only between *Hox2, Hox3* and *Hox4; Hox5* and *Hox6*; *Hox12* and *Hox13*^59,60^ (Fig. 3). In *O. dioica*, the total number of *Hox* genes is further reduced to eight, split between 6 scaffolds, including a duplication of *Hox9*^61,62^ (Fig. 3). In *M. oculata* we could identify only six *Hox* genes, divided between 4 scaffolds, with clustering retained for the *Hox10*, *Hox11* and *Hox12* genes (Fig. 3). In Botryllidae genomes, the same seven *Hox* genes are conserved (Fig. 3), with a preserved linkage between *Hox10*, *Hox12* and *Hox13* in *B. leachii* and three copies of *Hox5* present in *B. schlosseri*. Of the seven *B. leachii Hox* genes, transcripts for four are present in our reference transcriptome^37^ (*Hox1*, *Hox4*, *Hox10* and *Hox12*; File S5), indicating that they may still be functional. Two of the *Hox* genes (*Hox2* and *Hox5*) were not predicted by AUGUSTUS, nor were they present in the transcriptome (File S5); this may represent either partial (non-functional) genes, or a lack of expression in the tissues used to assemble the transcriptome. Altogether, the separation of the tunicate *Hox* cluster genes supports the hypothesis that reduction and separation of this ancient gene linkage occurred at the base of the tunicate lineage^61^. In addition, there is no particular pattern to the complement of retained *Hox* genes, with only *Hox1*, *Hox10* and *Hox12* being found in all five examined tunicate genomes (Fig. 3).

A second ancient linkage that we investigated is the pharyngeal cluster, a gene group present in hemichordates, echinoderms and vertebrate genomes that is considered to be Deuterosome specific^63^. The cluster groups *foxhead domain protein* (*FoxA*), *Nkx2 (Nkx2.2 and Nkx2.1)*, *Pax1/9*, *mitochondrial solute carrier family 25 member 21* (*slc25A21)*, *mirror-image polydactyly 1 protein* (*mipol1*), *egl nine homolog 3* (*egln3*) and *dehydrogenase/reductase member* 7 (*dhrs7*). Among these, s*lc25a21, Pax1/9*, *mipol1* and *FoxA* pairs are also found in protostomes suggesting an even more ancient origin^63^. The pharyngeal cluster is thought to have arisen due to the location of the regulatory elements of *Pax1/9* and *FoxA* within the introns of *slc25A21* and *mipol1*^64,65^, compelling these genes to remain physically located near each other in a genome.

**Figure 4:**
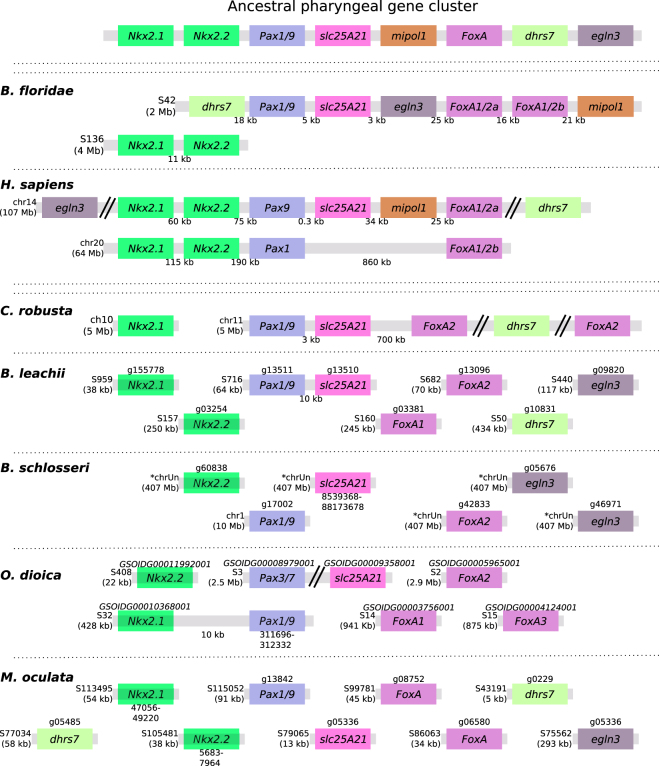
Ancestral gene linkages remain between a few pharyngeal cluster genes in tunicate genomes. Schematic depicting the organization of the pharyngeal cluster genes among the studied chordate genomes. Double-parallel lines indicate >1 Mb distance between genes. Chromosome (chr) or scaffold (S) number is shown, along with gene ID when available for newly annotated genomes. Orthologous genes are indicated by common colours. Transcript IDs for *B. leachii* genes identified in our transcriptome data^37^ are provided in File S5.

In the *B. floridae* genome, the entire cluster is located on the same scaffold, with the exception of the *Nkx2.1* and *Nk2.2* gene pair located on a separate scaffold, with an average intergenic distance of 14 kb (Fig. 4). In *C. robusta*, orthologs of *FoxA2*, *slc25a29*, *Pax1* and *dhrs7* are located on the same chromosome (chr 11, Fig. 4), with only *Pax1/9* and *slc25A29* located in close proximity to each other (~3 kb, Fig. 4). In *O. dioica*, orthologs of *FoxA*, *Pax1/9* and *Nkx2.2* genes were found on different scaffolds, with only one linkage (~10 kb), between *Pax1/9* and *Nkx2.1* genes, preserved. For both *B. schlosseri* and *M. oculata* there was no evidence of clustering between genes (Fig. 4). However, some *M. oculata* scaffolds are too small (5–13 kb) to make definite conclusions, given that the observed gene dispersal may be an artefact of a fragmented assembly. In the *B. leachii* genome, *mipol1* is the sole missing gene from this cluster and only the pairing of *Pax1/9* and *slc25A21* remains (Fig. 4).

**Figure 5:**
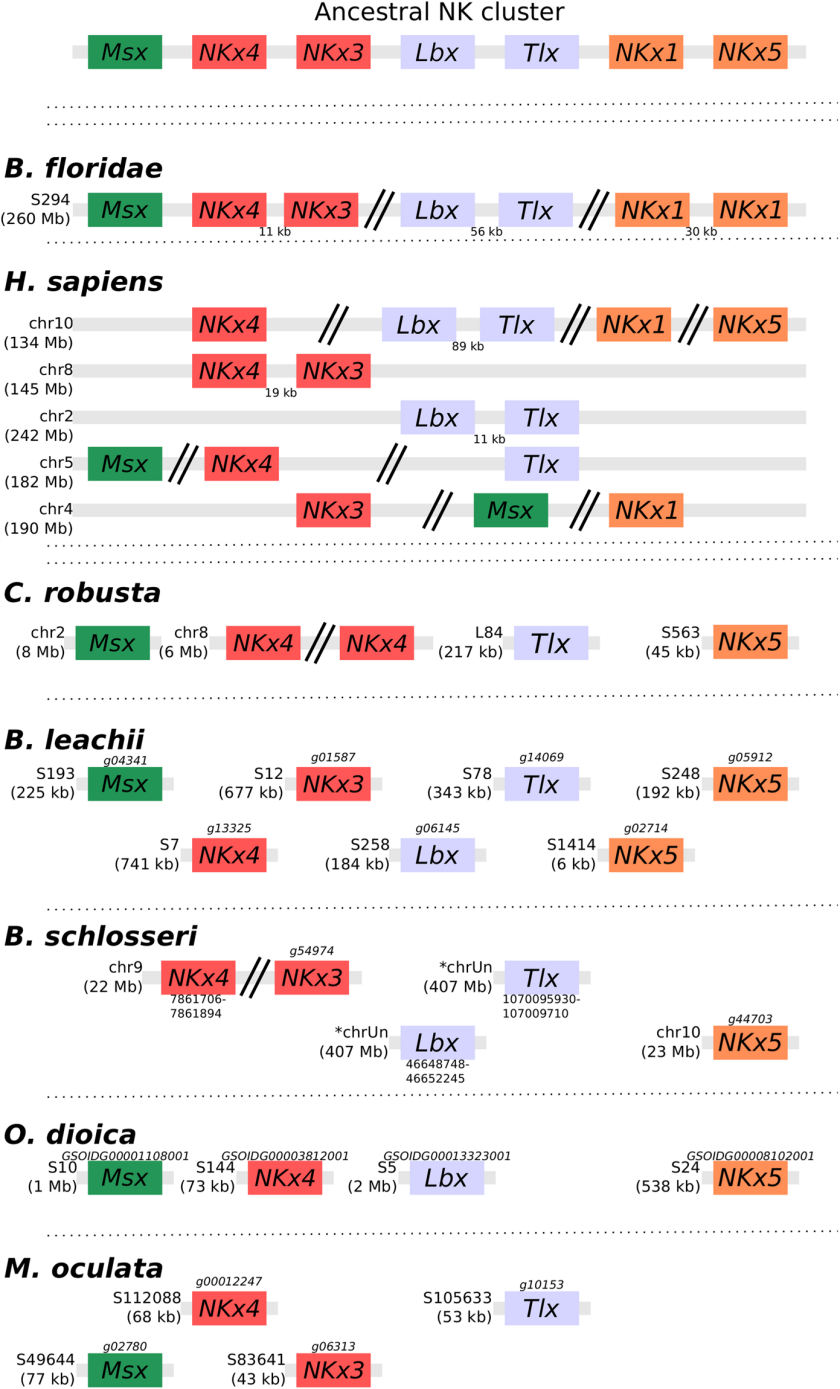
*NK homeobox* cluster genes are fragmented within tunicate genomes. Schematic depicting the organization of the *NK homeobox* cluster genes among the studied chordate genomes. Double-parallel lines indicate >1 Mb distance between genes. Chromosome (chr) or scaffold (S) number is shown, along with gene ID when available for newly annotated genomes. Orthologous genes are indicated by common colours. Transcript IDs for *B. leachii* genes identified in our transcriptome data^37^ are provided in File S5.

A third ancient homeobox-containing gene linkage is the *NK* cluster. This cluster, predicted to be present in the last common ancestor of bilaterians^66^, consists of *Msx, Lbx*, *Tlx*, *NKx1, NKx3, NKx4* and *NKx5* (Fig. 5). In *B. floridae*, linkages between *Msx, NKx4* and *NKx3*; as well as between *Lbx* and *Tlx* provide evidence of retained ancestral clustering while *NKx5* was lost^66^ (Fig. 5). However, in vertebrates, only the gene linkages between *Lbx* and *Tlx* as well as between *NKx4* and *NKx3* remain^55^ (Fig. 5). To further clarify the evolution of this ancestral cluster in tunicates, we determined the structure of the *NK* cluster within five ascidian genomes. In all these species, *NKx1* is absent and no evidence of clustering could be found with all identified orthologs located on different scaffolds or chromosomes (Fig. 5). While some of the assembled scaffolds are small (especially for *M. oculata*), even those tunicate genomes with assembled chromosome sequences and scaffolds larger than >1 Mb show no evidence of cluster retention, suggesting that most of the tunicates did not conserve the structure of this ancient linkage. In *M. oculata* only four members of this cluster were identified in the current assembly, with the loss of *NKx5* as well as *Lbx* (Fig. 5). In the colonial tunicates *B. leachii* and *B. schlosseri*, *Tbx*, *Lbx* and *NKx3* are all present. In *B. schlosseri*, *Msx* is absent and *NKx4* duplicated. In the *B. leachii* genome, *NKx1* is the only ancestral cluster member to be missing and *NKx5* has been duplicated (Fig. 5). These results suggest that there has been a loss of *NKx5* in cephalochordates, one of *NKx1* in tunicates and that the retention of both *Lbx* and *Tbx* may be specific to colonial ascidians. However, only *Msx* and *NKx4* were identified in our transcriptome (File S5), therefore we cannot be certain if *NKx3*, *Lbx1*, *Tlx* and *NKx5* genes are expressed and functional in *B. leachii*.

Taken together, these three results suggest that most of the tunicates did not conserve the structure of some ancient gene linkages. Further studies are needed to determine the consequences to both gene expression and function following loss of gene clustering.

### Lineage-specific changes to cell-signalling pathways in Botryllidae genomes

To examine the evolution of colonial ascidians more directly, we examined the genomes of *B. leachii* and *B. schlosseri*, looking for key components of signalling pathways required for metazoan development and regeneration. Of particular interest, we focused on the Wingless-related integration site (Wnt), Notch and Retinoic acid (RA) signalling pathways. All three of these pathways have been implicated in WBR and asexual reproduction in colonial tunicates^25,37,67^.

### Wnt pathway

Wnt ligands are secreted glycoproteins that have roles in axis patterning, morphogenesis and cell specification^68^. The ancestral gene family appears to have originated early during multi-cellular evolution and is composed of eleven members^69,70^. The *Wnt* gene family expanded to 19 members in the human genome, while independent gene loss has reduced this family to 7 genes in *Drosophila melanogaster* and *Caenorhabditis elegans*^71^. Consequently, we investigated whether the *Wnt* gene family has either expanded or contracted during Tunicata speciation.

**Figure 6:**
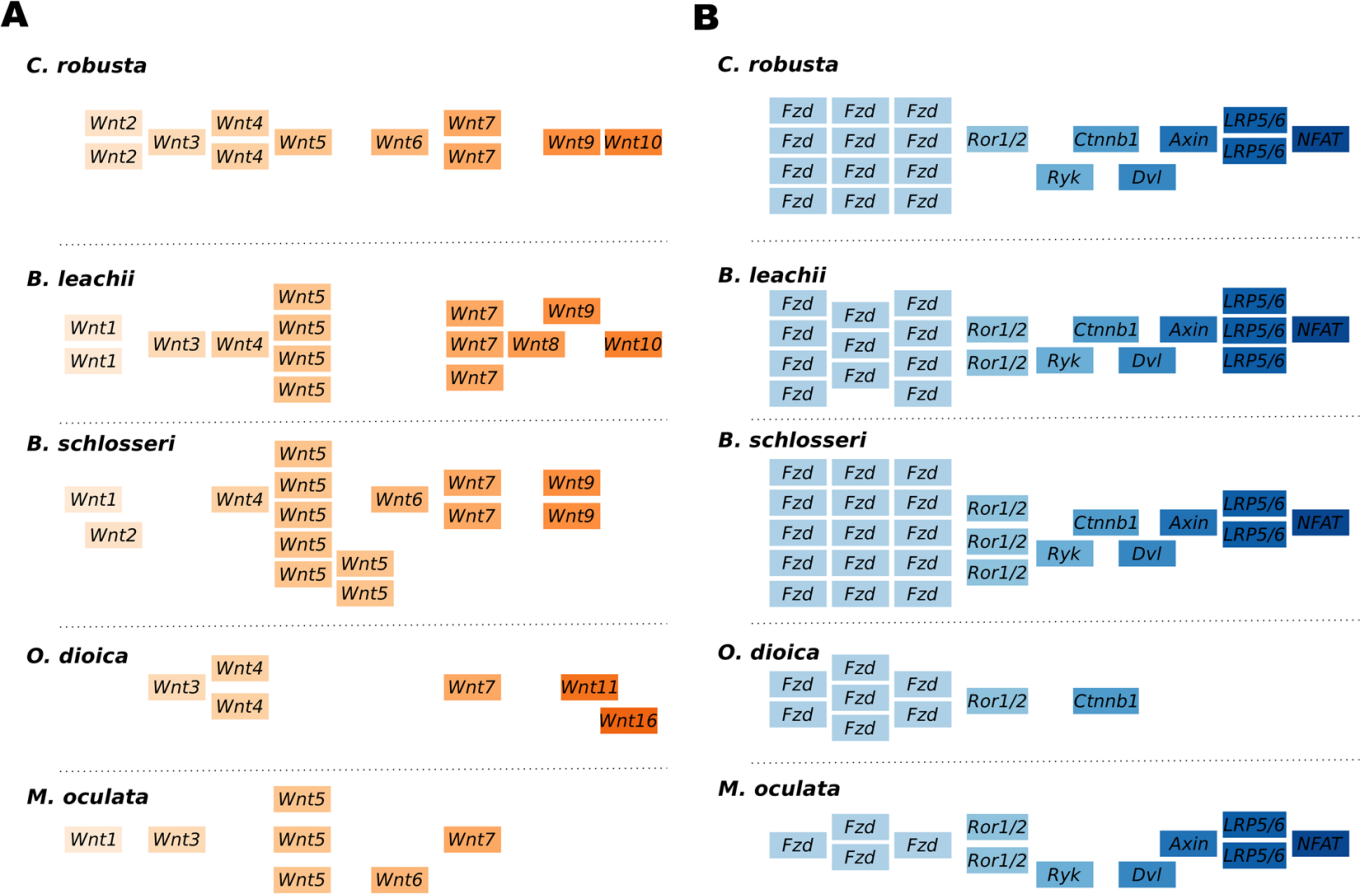
Duplication of components of the *Wnt* signalling pathway in tunicate genomes. Schematic showing the organization of (**A**) the *Wnt* genes within each indicated genome and (**B**) of the downstream effectors. Note that no *Wnt5* ortholog is present in the *O. dioica* genome. Genome browser images for the *Wnt5* genes are shown in Fig. S4.

We found an increase in the number of *Wnt5a* genes among Styelidae genomes (Fig. 6A). In *B. schlosseri*, we identified 15 *Wnt* members, including seven *Wnt5a* genes on multiple scaffolds (Fig. 6A). In the *B. leachii* genome, fourteen *Wnt* ligand genes were identified, including four *Wnt5a* genes located on the same scaffold near *Wnt4* (Fig. S3). *M. oculata* has only 7 *Wnt* ligand genes, including three *Wnt5a* genes (Fig. 6A). In comparison, *C. robusta* has a total of 11 *Wnt* genes, including a single copy of *Wnt5a*^60^ (Fig. 6A). In the compact *O. dioica* genome, this number is reduced to 6 (*Wnts* 3, 4, 7, 11 and 16), none of which are *Wnt5a* orthologs (Fig. 6A). The various orthologs of the duplicated *Wnt5a* genes show a similar exon-intron structure (Fig. S3), which indicates that they are likely to have arisen through gene duplication^72^. Overall, our data suggests that an expansion, possibility through gene duplication, of the *Wnt5a* family occurred during tunicate evolution, but was lost in some lineages.

To assess the functionality of the Wnt pathway in tunicates, we evaluated whether its downstream effectors are also present in the available genomic data. The downstream pathways activated by Wnt ligands are divided into canonical, non-canonical calcium and non-canonical planar cell polarity. The Wnt5a ligand is associated with both of the non-canonical pathways through binding of membrane receptors that include frizzled (Fzd4), receptor tyrosine kinase-like orphan receptor 1/2 (Ror1/2) and atypical tyrosine kinase receptor (Ryk)^68^. Further downstream, dishevelled (Dvl), β-catenin (Cnntb), Axin, low-density lipoprotein receptor-related protein 5/6 (LRP5/6) and nuclear factor of activated T-cells (NFAT) are proteins essential for triggering intracellular responses to Wnt signalling^73^. We identified orthologs for each of these signalling transduction molecules in all Tunicata genomes (Fig. 6B), with no evidence of further gene duplication events. Importantly, 90% of the genes identified in *B. leachii* (35/39) have a corresponding transcript in the transcriptome^37^ (File S5). This supports the interpretation that signalling through the Wnt pathway is functional in tunicates.

### Notch pathway

Notch receptors are transmembrane proteins that are involved in cell-cell signalling during development, morphogenesis and regeneration^74^. Following activation through the binding of the Delta or Jagged/Serrate ligands, the intracellular domain of Notch is cleaved and induces the expression of downstream target genes including the *hairy and enhancer of split (hes*) gene family members^75^. The presence of both Notch and the Delta/Serrate/lag-2 (DSL) proteins in most metazoan genomes suggests that their last common ancestor had a single copy of each gene^76^. To establish how this pathway has evolved in tunicates, we screened these genomes for the Notch receptor using the conserved Lin12/Notch Repeat (LNR) domain, as well as for genes encoding probable Notch ligands.

**Figure 7:**
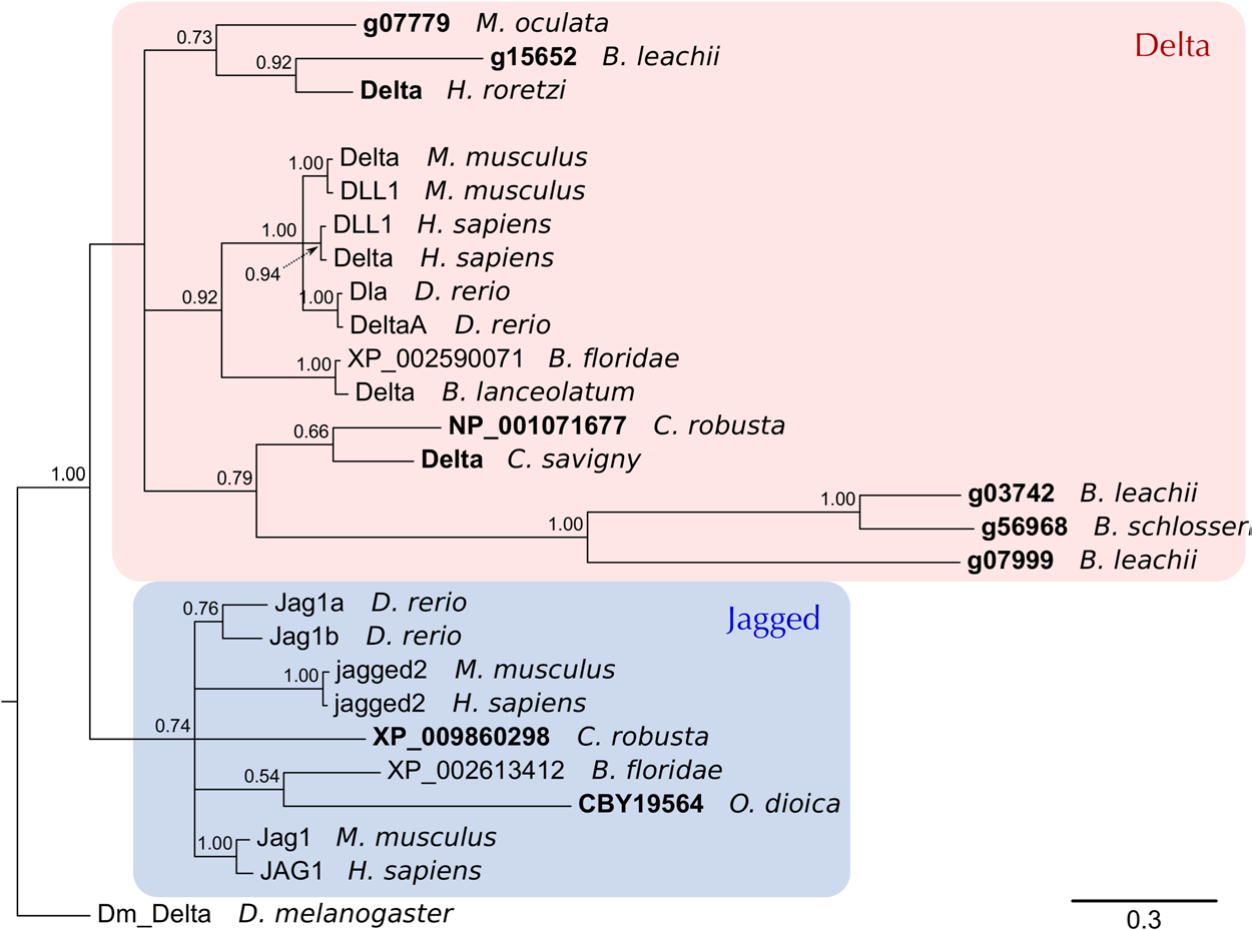
Tunicate Delta proteins. Bayesian phylogenetic tree depicting the relationship between tunicate and vertebrate DSL proteins, using *Drosophila* Delta to root the tree. Tunicate proteins are shown in bold and shaded areas correspond to Delta and Jagged groupings. Branch support values (probabilities) are indicated.

In all examined genomes, only a single *Notch* receptor gene was identified while the number of ligand genes varied (Fig. S4A). The *C. robusta* genome contains two *DSL* genes, while *O. dioica, M. oculata* and *B. schlosseri* possess only a single *DSL* gene. By contrast, we found three DSL genes in *B. leachii* (Fig. S4A). To determine the relationships between these identified tunicate DSL-like genes, a phylogeny was constructed with other chordate DSL proteins. All three *B. leachii* genes are Delta orthologs, two of them related to the *B. schlosseri* and *Cionidae* proteins; the third one closer to the *M. oculata* and *H. roretzi* variants. The mouse, human and zebrafish delta and delta-like (DLL) proteins form a discrete clade loosely related to the genes found in cephalochordates and tunicates (Fig. 7, red box). Jagged proteins form a separate clade where no subphylum-specific clustering is observed (Fig. 7, blue box). The tunicate DSL-like proteins show long phylogenetic branches, suggestive of greater diversity, also observed in the protein alignment (Fig. S5A). This suggests that the tunicate DSL proteins are diverging rapidly from each other, indicative of lineage specific evolution of DSL-like genes.

### Retinoic acid signalling

**Figure 8:**
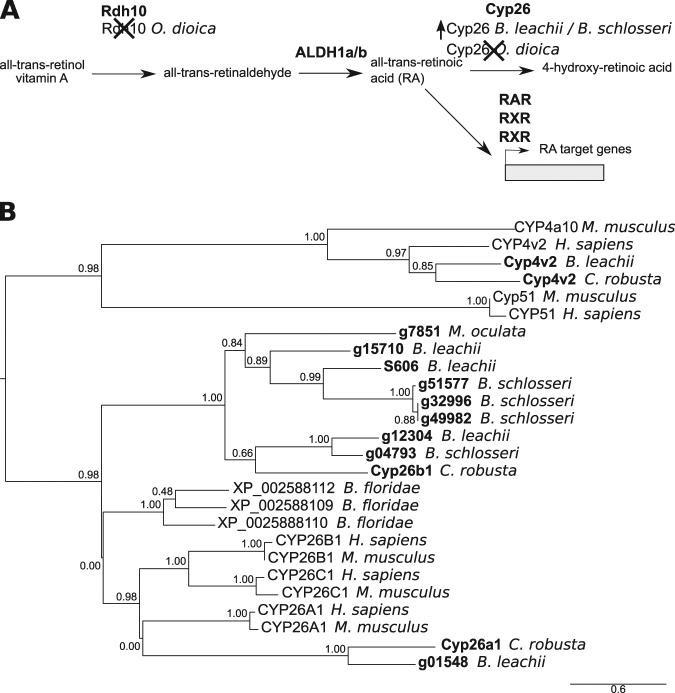
Evolution of the RA pathway in tunicates. (**A**) Overview of the RA synthesis and degradation pathway. In bold are the major proteins that contribute to RA signalling during animal development. Indicated below these are changes to the number of copies present in examined genomes. (**B**) Maximum likelihood phylogenetic tree depicting the relationship between invertebrate and vertebrate CYP26 proteins using CYP4 and CYP51 proteins as an out-group. Tunicate proteins are shown in bold. No *Cyp26* gene has been identified in the *O. dioica* genome^81^. Values for the approximate likelihood-ratio test (aLRT) are indicated.

Retinoic acid (RA) is an extracellular metabolite that is essential for chordate embryonic development. RA is synthesized from retinol (vitamin A) by two successive oxidation steps. In the first step, retinol dehydrogenase (Rdh) transforms retinol into retinal. Then RA is produced by aldehyde dehydrogenase (Aldh), a superfamily of enzymes with essential roles in detoxification and metabolism^77^. RA influences the expression of downstream target genes by binding to the RA receptors, RAR and RXR^78^ (Fig. 8A). Finally, RA is metabolized by the cytochrome P450 family 26 (Cyp26) enzyme, which absence of expression can restrict RA-induced responses to specific tissues or cell types^79,80^. Components of this pathway have been found in non-chordate animals, suggesting a more ancient origin^80^. This pathway is required for *B. leachii* WBR^25^ and *Ciona* development, yet several genes essential for RA signalling appear to be missing in *O. dioica*^80,81^.

Rdh10 is the major dehydrogenase associated with the first steps of RA production, although the Rdh16 and RdhE2 enzymes can also substitute this function^82–84^. The *O. dioica* genome has no orthologs for either *Rdh10* or *Rdh16* but it does have four genes that encode for RdhE2 proteins^81^. *O. dioica* also lacks both an *Aldh1-*type gene, as well as a *Cyp26* gene, but has one single RXR-ortholog^81^ (Fig. S5B). In contrast, the *C. robusta* genome contains single copies of *Rdh10*, *Rdh16* and *RdhE2* genes and a total of four *Aldh1* genes, located on two chromosomes^80^. Consistent with *C. robusta*, *M. oculata*, *B. leachii* and *B. schlosseri* genomes all have single copies of *Rdh10, Rdh16* and *RdhE2* genes, as well as three *Aldh1* genes on separate scaffolds (Fig. S5B).

Three retinoic acid receptor genes were identified within the *B. leachii* genome, one of which had been previously cloned^25^ (*g03013* in our assembly; File S5). All three were also found in *C. robusta*, *M. oculata* and *B. schlosseri* genomes (File. S5B). While there is only one potential *Cyp26* gene in *M. oculata*, four paralogs were identified in *B. leachii* and *B. schlosseri*. A phylogenetic analysis, using related chordate Cyp proteins as an outgroup, showed that these paralogs cluster with Cyp26 proteins (Figs 8B and S5B). Altogether, these results show a loss of key RA-pathway genes in *O. dioica* (*Rdh10, Rdh16, Cyp26* and *Aldh1*) while, in non-larvacean tunicates, the copy numbers of some genes has increased, suggesting that RA signalling pathway is still functional.

## Discussion

### Genomic diversity within the Stolidobranchia

The diversity of changes to developmental pathways observed between the *B. leachii* genome and that of closely related ascidians, along with previous genomic analyses of other ascidian species, supports the widely held view that ascidian genomes are diverse and rapidly evolving, which is particularly evident in the Stolidobranchia group^5,13–15,85–88^. Nevertheless, Styelidae were sufficiently similar in external appearance and morphology for early researchers to suggest that *Botrylloides* could be a subgenus of *Botryllus*^89,90^. Strikingly however, the *B. schlosseri* genome differs from that of *B. leachii*, as well as from other sequenced tunicate genomes (Table 1). The main genomic differences between *B. leachii* and *B. schlosseri* are in their genome sizes (194 Mb vs 725 Mb), their fraction of repetitive sequences (19% vs 60%^15^) and the number of predicted genes (15,839 vs 27,463^15^). The genome size of *B. schlosseri* resembles more that of the Thaliacean *S. thompsoni*^7^ than that of *B. leachii* (*S. thompsoni* genome size: 602 Mb, repetitive sequences: 60–70%, genes: 26,415; Table 1). Altogether, these comparisons indicate that genome expansion is not necessary for coloniality in ascidians, and that the *B. schlosseri* genome has an architecture divergent from that of *B. leachii* by having undergone a significant increase in its genomic content, including repetitive element expansion (File S2).

Rapid genome evolution, and active transposable elements in particular, are proposed to aid adaptation to new environments for invasive species^91^. Differences have been noted in the range of tolerable environmental conditions, such as salinity or temperature, which permits the colonization of a given habitat by tunicates, not only between *B. leachii* and *B. schlosseri*^92–94^, but even within the *B. schlosseri* cryptic species complex^87,90^. It is possible that such plasticity in genome characteristics, like transposon diversity, genome size and gene number, assists the observed invasive success of tunicate species^95^.

Ancient homeobox genes clusters whose structure has been retained over millions of years of evolution in many organisms appear fragmented in the available tunicate genomes. Because the expression of each *Hox* gene across the anterior-posterior axis relates to their genomic location within the *Hox* gene cluster^57^, cluster breaks are predicted to have consequences for patterning processes. However, an adult body plan with correct spatial orientation of its body axes is also established during sexual and asexual development, including WBR, in ascidians. Embryonic patterning events in tunicate species have only been well characterized during *Ciona* sexual reproduction. Early stages of development (prior to gastrulation) follow a mosaic pattern of developmental axis formation, where inheritance of maternally provided factors establishes the body axes^96^. *Hox* gene knockdown experiments in *C. robusta* revealed that *Hox* genes have very limited roles, with defects only observed in the development of the neurons and tails of the larvae^97^. Therefore, it appears that embryonic patterning events in *C. robusta* are not dependent upon *Hox* genes function to establish regional identity. However, *Hox* genes do play a role later on, during metamorphosis where knockdown of *Hox1*, *Hox10* and *Hox12* causes tissue malformation and adult death^97,98^, while no functions have been attributed to the other Hox genes^98^. These three posterior *Hox* genes are the ones present in all studies tunicate genomes (Fig. 3). Thus, it will be of interest to determine the consequences of *Hox* cluster dispersion and gene loss to the formation of adult organs during sexual and asexual reproduction in colonial ascidians. In animals many mechanisms, in addition to molecular factors, act to establish regional tissue patterning^99^. In *B. schlosseri*, the entry point of the connective test vessel into the developing bud determines the posterior end of the new zooid^100^. Therefore, we hypothesize that physical and/or environmental cues could help compensate for the loss of *Hox* gene function in determining regional identity during asexual development. A wider analysis comprising multiple tunicate species will be necessary to investigate the exact consequences of homeobox cluster dispersion and compensatory mechanisms.

These three examples highlight the genomic diversity which exists among tunicates, and within the Stolidobranchia in particular. These organisms provide a unique opportunity to study the functional impact of such genomic variations by comparing closely related species.

### Gene orthology analysis and innovations

As a first step towards investigating the genetics underlying tunicate biology, we have performed an all-to-all search for protein orthologs between five tunicate species (File S3). Among the tunicate orthologous clusters that we obtained, we identified several groups of genes that are not shared by all the tunicate genomes (Fig. 2A). Of particular interest are genes found only in the *B. schlosseri* and *B. leachii* genomes, as these may function in biological processes unique to colonial tunicates. Many of these genes have orthologs not only in vertebrates, but also in more evolutionarily distant animals such as *C. elegans* (File S4). This suggests that these genes have a more ancient origin, which was retained specifically in Botryllidae genomes. The overrepresented biological processes include circulation, wound healing and cell communication; as well as regulation of immune cell differentiation and immune system processes. Unlike solitary tunicate species, colonial ascidians share a complex system of single cell-lined vessels, used to transport haemocytes and facilitate communication between zooids within the colony, that is the exclusive site of WBR following zooid loss^101^. In addition, immune response is known to have roles in wound healing, vasculogenesis, allorecognition and regeneration^102–104^. Therefore, it is possible that these genes, found only in *Botryllus* and *Botrylloides*, contribute to biological pathways and cellular processes that have important roles in colonialism. Expansion of ortholog analysis to include additional genomes from other newly sequenced tunicates will further refine the set of candidate genes belonging to these processes. For instance, including the Thaliacean *S. thompsoni* (which has both colonial and solitary life stages) would be of interest for studying colonialism, while incorporating regenerating Phlebobranchian species such as *Perophora viridis*^105,106^ would help identify genes involved in regeneration.

Both *O. dioica* and *B. schlosseri* had a high number (2160 and 2716 respectively) of clusters unique to their genomes (Fig. 2A). While the *O. dioica* genome has undergone considerable loss of ancestral genes^13,107^, the total number of genes in this species is similar to that of other tunicates (Table 2). Taken together, these observations suggest that there has been a duplication of the retained genes such as *Otx* (3 copies in *O. dioica*, one in *Ciona*^108^). The *B. schlosseri* genome has an ~10,000 higher predicted gene number compared to other tunicates (Table 2), suggesting partial genome duplication. Further analysis will be required to determine whether these are novel or duplicated genes, hence providing important insights in the evolution of Tunicata genomes.

### Lineage-specific changes to evolutionarily conserved cell communication pathways

Cell signalling pathways are critical for morphogenesis, development and adult physiology. We have focused our analysis on three highly conserved pathways: Wnt, Notch and RA signalling. Representatives of all twelve *Wnt* gene subfamilies are found in metazoans, suggesting that they evolved before the emergence of the bilaterians^109^. We identified members of each Wnt subfamily among the studied tunicate genomes, along with numerous examples of lineage-specific gene loss and/or duplication. The most striking was an increase in *Wnt5a* gene copy number in *B. leachii*, *B. schlosseri* and *M. oculata*. Indeed, most invertebrate genomes, including the basal chordate *B. floridae*, contain a single *Wnt5a* gene while most vertebrate genomes have two *Wnt5a* paralogs, believed to be a result of whole genome duplication^110^. Potentially, these additional genes have been co-opted into novel roles and were retained during tunicate evolution. Wnt5a ligands have numerous biological roles, including a suppressive one during zebrafish regeneration^111^ and a promotive one during amphioxus regeneration^112^. Components of both Wnt signaling pathways are differentially expressed during *B. leachii* WBR^37^, it is possible that *Wnt5a* gene number has expanded in colonial tunicates to sustain WBR. The functional characterization of *Wnt5a* genes during *B. leachii* WBR will be explored in future studies.

All components of the Notch pathway are present in the genomes we investigated. Of particular interest, the DSL Notch ligand appears to be rapidly evolving in tunicates. This indicates that tunicate DSL proteins are under less pressure to conserve their sequence than their vertebrate orthologs. Given that the interaction between the DSL domain and the Notch receptor is central to signalling pathway activation^113^, it will be interesting to assess whether the functional ligand-receptor interactions between tunicate DSL proteins and tunicate Notch proteins have adapted accordingly.

Components of the RA signalling pathway have also been identified in all the tunicate genomes. However, *Oikopleura* has seemingly lost a functional RA synthesis pathway, while still forming a functional body plan. This suggests that *O. dioica* utilizes an alternative synthesis approach, that the RA signalling function has been replaced or, and rather uniquely, that RA is not involved in the development of this species. Conversely, lineage specific increases in RA pathway gene numbers have been observed in *C. robusta*^114^ (Aldh1) and *Botrylloides* (*Cyp26* genes, Fig. 8), suggestive of a functional role at some stage of their development.

RA, Notch and Wnt pathways play roles in regeneration and development in many species, including Stolidobranchian tunicates^25,37,67^ and *Cionidae*^19,74^. The involvement of such conserved signalling pathways opens a number of interesting hypothesis. While the regenerative potential of *O. dioica* has not been characterized, the observed loss of RA signalling genes may implicate a reduced regeneration ability compared to the other tunicates. Given the unique WBR potential developed by colonial tunicates, the selective pressure on their genomes to retain these pathways might be higher than that of other chordates. Additionally, because of the morphological similarities between WBR and colony reactivation following hibernation, it appears plausible that these three pathways play a similar role in these processes.

Among tunicates, even between closely related species, there exist significant differences in life cycle, reproduction and regeneration ability, which likely reflect an underlying diversity in genomic content. For instance, differences in both asexual and sexual reproduction have been observed within the Botryllidae family^19,35,92,93^. Furthermore, *B. schlosseri* can only undergo WBR during a short time frame of its asexual reproductive cycle when the adults are reabsorbed by the colony^8,115^ while *B. leachii* can undergo WBR throughout its adult life^116^. Overall, this indicates that despite a generally similar appearance, the rapid evolution of the Tunicata subphylum has provided diversity and innovations within its species. In future studies, it will be interesting to investigate how such genomic plasticity balances between adaptation to new challenges and constraint, preserving common morphological features.

### Summary

In conclusion, our assembly of the *B. leachii* genome provides an essential resource for the study of this colonial ascidian as well as a crucial point of comparison to gain further insights into the remarkable genetic diversity among tunicate species. In addition, the genome of *B. leachii* will be most useful for dissecting WBR in chordates; particularly through comparison with *B. schlosseri* for understanding how the initiation of WBR can be blocked during specific periods of its life cycle. Furthermore, given the key phylogenetic position of tunicates with respect to vertebrates, the analysis of their genomes will provide important insights in the emergence of chordate traits and the origin of vertebrates.

## Methods

### Sampling, library preparation and sequencing

*B. leachii* colonies were collected from Nelson harbour (latitude 41.26°S, longitude 173.28°E) in New Zealand. To reduce the likelihood of contamination, embryos were dissected out of a colony and pooled before carrying out DNA extraction using E.Z.N.A SP Plant DNA Mini Kit (Omega Biotek). A total of 4 µg DNA was sent to New Zealand Genomics Limited (University of Otago, NZ) for two runs of library preparation and sequencing. Short read sequencing of Illumina TruSeq libraries in a HiSeq. 2500 generated 19,090,212 paired-end reads of 100 bp (average fragment size: 450 bp, adaptor length: 120 bp). A second sequencing (Illumina Nextera MiSeq Mate Pair), without size-selection, generated 31,780,788 paired-end sequences of 250 bp (fragment size: 1.5–15 kb, median size: ~3 kb, adaptor length: 38 bp).

Pre-quality check report was generated using the String Graph Assembler software package^38^ and quality metrics before assembly with both FastQC^117^ as well as MultiQC^118^. These analyses revealed that 91% of sequences had a mean Phred quality score >=30, 96% a mean Phred quality score >=30 and 39% of sequences had an adapter sequence (either Illumina or Nextera, Fig. S1). Adaptor trimming was performed with NxTrim^119^ for the mate pair library, followed by Trimmomatic^120^ with the following options: MINLEN:40 ILLUMINACLIP:2:30:12:1:true LEADING:3 TRAILING:3 MAXINFO:40:0.4 MINLEN:40 for both libraries. After trimming, 86,644,308 paired-end (85%) and 12,112,004 (12%) single-end sequences remained (100% with a mean Phred quality score >=30, <1% with an adapter sequence, Fig. S1).

### Genome assembly

*De novo* assembly was performed in three consecutive iterations following a Meta-assembly approach (Table S5). First, both libraries were assembled together in parallel, using a k-mer size of 63 (when available) following the results from KmerGenie^121^, by five assemblers: ABySS^122^, Velvet^123^, SOAPdenovo2^124^, ALLPATHS-LG^125^, MaSuRCA^126^. The MaSuRCA assembler was run twice, once running the adapter filtering function (here termed “MaSuRCA-filtered”), the other without it (termed simply “MaSuRCA”). Their respective quality was then estimated using three different metrics: the N50 length, the BUSCO core-genes completion^42^ and the number of genes predicted by Glimmer^127^. Second, these drafts were combined by following each ranking using Metassembler^41^, hence producing three new assemblies (limiting the maximum insert size at 15 kb). Third, the *B. leachii* transcriptome^37^ was aligned to each meta-assembly using STAR^128^, and their alignment percentage was used as ranking in a third run using Metassembler, limiting the maximum insert size at either 3 kb, 8 kb or 15 kb. Finally, the quality of the meta-meta-assemblies was estimated using the BUSCO score and the best one (Table S5) selected as the reference *de novo* assembly.

### Data access

All data was retrieved from the indicated sources in January 2016. Note that *Ciona intestinalis* type A^14^ has recently been recognized as a distinct species (*Ciona robusta*^129^).

*B. schlosseri*, *C. robusta*, *M. oculata*: Ascidian Network for *In Situ* Expression and Embryonic Data (ANISEED, https://www.aniseed.cnrs.fr/aniseed/).

*O. dioica*: Oikopleura Genome Browser (http://www.genoscope.cns.fr/externe/GenomeBrowser/Oikopleura/).

*B. floridae*, *H. sapiens*: Joint Genome Institute (JGI, http://genome.jgi.doe.gov).

Quality assessment comparison data for *Homo sapiens*, *Maylandia zebra, Melopsittacus undulatus, Crassostrea gigas*: String Graph Assembler (https://github.com/jts/sga/tree/master/src/examples/preqc).

The data of the *B. leachii* genome is available from the following sources:

Raw sequence reads: National Center for Biotechnology Information (NCBI) Sequence Read Archive (SRA, https://www.ncbi.nlm.nih.gov/sra) with the accession number SRP127769.

Assembled and annotated genome, predicted transcriptome, predicted proteome, species-specific AUGUSTUS and SNAP models: ANISEED (https://www.aniseed.cnrs.fr/aniseed/).

### Repeat region analysis

A *de novo* repeat library was built for each tunicate genome using RepeatModeler^50^. This utilizes the RECON tandem repeats finder from the RepeatScout packages to identify species-specific repeats in a genome assembly. RepeatMasker^49^ was then used to mask those repeats. This repeat library is available on ANISEED (https://www.aniseed.cnrs.fr/aniseed/).

### Gene annotation

*Ab initio* genome annotation was performed using MAKER2^44^ with AUGUSTUS^130^ and SNAP^131^ for gene prediction. In addition, we used our previously published transcriptome^37^ and a concatenation of UniProtKB^132^, *C. robusta* and *B. schlosseri* proteins into a custom proteome as evidence of gene product. Using the predicted genes, AUGUSTUS and SNAP were then trained to the specificity of *B. leachii* genome. A second round of predictions was then performed, followed by a second round of training. The final annotation of the genome was obtained after running a third round of predictions, and the provided trained AUGUSTUS and SNAP configurations after a third round of training. Non-coding RNA sequences were annotated using Infernal^46^ with the Rfam library 12.0^133^, tRNAscan-SE^48^ and snoRNA^47^. Finally, the identified sequences were characterized by InterProScan^45^.

### Analysis of Gene Ontology terms

Distribution of Gene Ontology (GO) terms were computed for each species as follows. GO terms were extracted from the genome annotation and the number of occurrence for each term determined using a custom Python script. The resulting list of frequencies was then simplified using REVIGO^134^ (similarity factor “small” of 0.5) and the TreeMap output retrieved. The hierarchy of every GO term present was reconstructed following the schema defined by the core gene ontology (go.obo^135^) using a custom Python script selecting the shortest path to the root of the tree, favouring smaller GO terms identification number in case of multiple paths. Finally, frequencies were displayed using the sunburstR function of the Data-Driven Documents library^136^ (D3).

Predicted amino acid sequences for all species were retrieved and clustered into 17,710 groups by OrthoMCL^52^. Protein sequences within each group were then aligned into a Multiple Sequence Alignment by Clustal-Omega^137^, and the corresponding consensus sequence inferred by cons (EMBOSS^138^). Consensus sequences were matched to the Swiss-Prot curated database using BLASTp^51^ (e-value cut-off of 10^−5^), and the GO terms corresponding to the best match retrieved. GO terms frequencies were analysed as described above and displayed using REVIGO and TreeMap. Results from OrthoMCL (groups of clustered genes), Clustal-Omega (multiple sequence alignments), EMBOSS (consensus sequences) and BLASTp (top hit) are provided in File S3.

The overrepresentation analysis was performed using GOrilla^139^ with *Homo sapiens* as the organism background, using a *p*-value threshold of 10^−3^ and REVIGO TreeMap (similarity factor “medium” of 0.7) for visualization. Results are provided in File S4.

### Analysis of specific gene families

Genes and transcripts for each examined genome were identified by a tBLASTn search (e-value cut-off of 10^−5^) using the SequencerServer software^140^. This was followed by a reciprocal BLAST using SmartBLAST^141^, to confirm their identity.

Conserved protein domains used to identify the corresponding orthologous proteins within tunicate genomes are found in Table S2.

### Phylogenetics

Sequences were aligned with ClustalX^142^ before using ProtTest 3^143^ to determine the best-fit model of evolution. The best-fit models for the DSL and CYP26 phylogeny were WAG + I + G and LG + I + G, respectively.

Bayesian inference (BI) phylogenies were constructed using MrBayes^144^ with a mixed model for 100,000 generations and summarized using a Sump burn-in of 200. Maximum Likelihood (ML) phylogenies were generated by PhyML^145^, using the estimated amino acid frequencies.

Accession numbers are provided in File S5 and sequence alignments are provided in Fig. S5. Analyses carried out with BI and ML produced identical tree topologies. Trees were displayed using FigTree v1.4.2 (http://tree.bio.ed.ac.uk/software/figtree/).

## Electronic supplementary material

Supplementary Figure Legends and files

Supplementary Dataset 1

Supplementary Dataset 2

Supplementary Dataset 4

Supplementary Dataset 5
