# Supplementary material for: De novo draft assembly of the Botrylloides leachii genome provides further insight into tunicate evolution
Source: Sci Rep. 2018 Apr 3;8:5518. doi: 10.1038/s41598-018-23749-w (PMC5882950; doi:10.1038/s41598-018-23749-w)
Supplement: Supplementary file 1 — Supplementary Figure Legends and files [file 41598_2018_23749_MOESM1_ESM.pdf]

De novo draft assembly of the *Botrylloides leachii* genome provides further insight into tunicate evolution.

Simon Blanchoud, Kim Rutherford, Lisa Zondag, Neil J. Gemmell and Megan J. Wilson

Supplementary Figures

Figure S1. *B. leachii* has a genome well suited for *de novo* assembly. (**A**) Quality check results for the sequencing libraries. (**B**) de Bruijn graph quantification presenting the computational complexity of the assembly.

Figure S2. Gene Ontology terms identified in the orthologs clusters (**A**) shared by colonial botryllids, (**B**) shared by all analyzed tunicates, (**C**) shared by the sessile tunicates, (**D**) specific to *B. schlosseri* and (**E**) specific to *O. dioica*. The names of the species included in a given group are written above each REVIGO table.

Figure S3. Duplication of *Wnt5a* genes in tunicate genomes. Genomic representation of the intron-exon structure of *Wnt5-like* genes within each indicated genome. Note that no *Wnt5a* ortholog is present in the *O. dioica* genome. Major ticks indicate 1 Mb, minor ticks 100 bp and double-parallel lines a gap of > 1 Mb.

Figure S4. Tunicate genes for (**A**) the Notch signalling pathway and (**B**) the Retinoic Acid signalling pathway.

Figure S5. Protein sequence alignments used to generate the phylogenies presented in the main text. (**A**) Alignment for the Notch signalling pathway shown in Fig. 7. (**B**) Alignment for the Retinoic Acid signalling pathway shown in Fig. 8.

Supplementary Files

File S1. BUSCO results for the *B. leachii* genome.

File S2. Repetitive elements identified by RepeatMasker using *de novo* repeat libraries for the genomes of *B. leachii*, *B. schlosseri*, *C. robusta*, *O. dioica* and *M. oculata*.

File S3. Zip folder containing the results from the tunicate orthologs analysis including gene clustering, multiple sequence alignments, consensus protein sequences and BLASTp hits. A README.txt file provides further details on the content of each file. This is available to download at http://wilsonlab.otago.ac.nz/projects/genome.

File S4. GOrilla and REVIGO results used for GO term overrepresentation analysis in the orthologs group between the *B. leachii* and *B. schlosseri* genomes.

File S5. Gene IDs and corresponding transcript IDs (see Zondag *et al.*, 2016) for genes of interest. This table includes accession numbers for the protein sequences used in phylogeny construction (Fig. 7 and 8).

Supplementary Tables

Table S1. Quality metrics of the iterative meta-assembly approach followed for the *de novo* assembly of the *B. leachii* genome.

Table S2. Conserved domains used to identify the corresponding proteins in tunicate genomes.

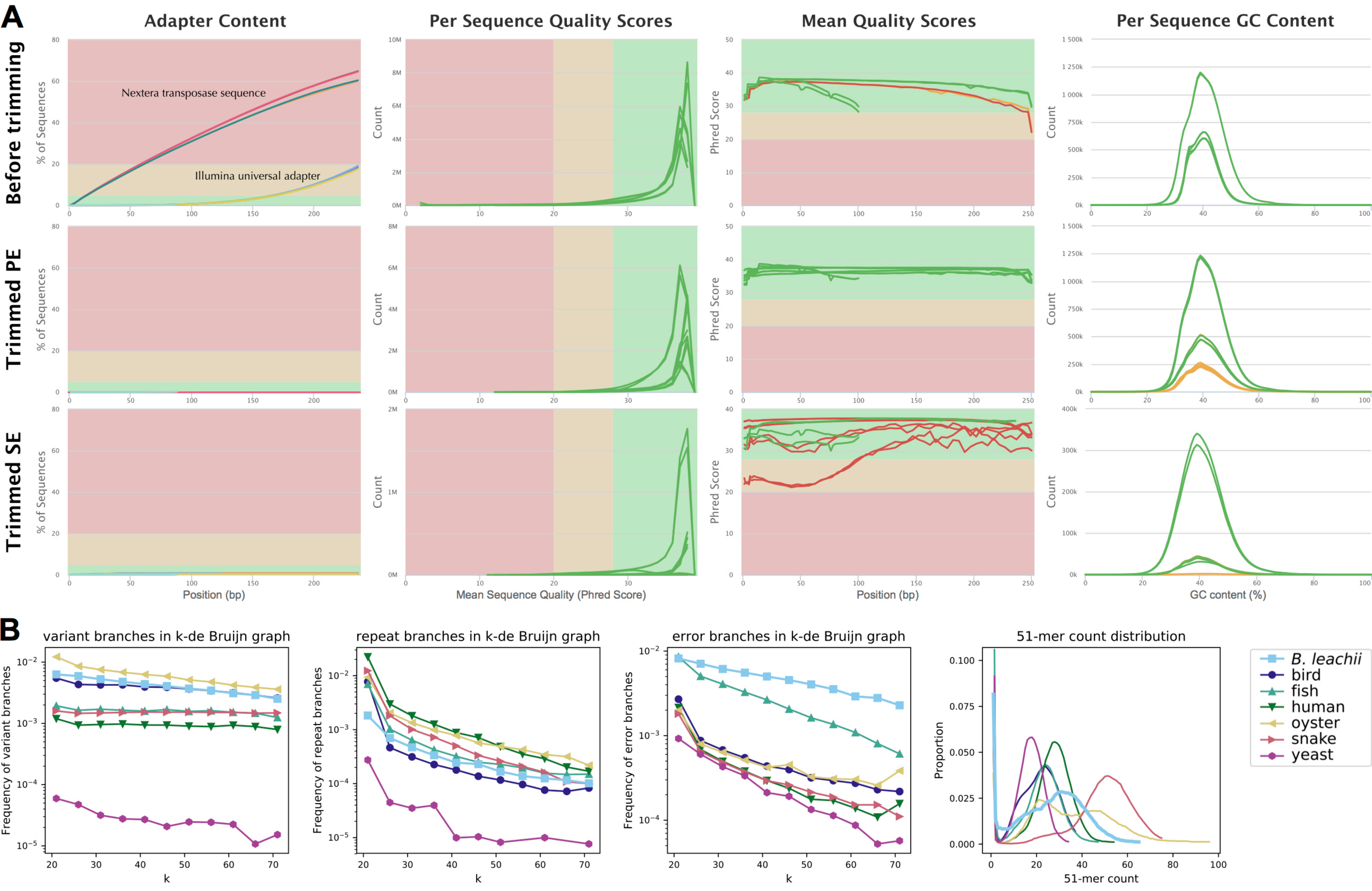
A
Adapter Content
Per Sequence Quality Scores
Mean Quality Scores
Per Sequence GC Content
Before trimming
Trimmed PE
Trimmed SE
% of Sequences
Count
Phred Score
Nextera transposase sequence
Illumina universal adapter
Position (bp)
Mean Sequence Quality (Phred Score)
GC content (%)
B
variant branches in k-de Bruijn graph
repeat branches in k-de Bruijn graph
error branches in k-de Bruijn graph
51-mer count distribution
Frequency of variant branches
Frequency of repeat branches
Frequency of error branches
Proportion
k
51-mer count
B. leachii
bird
fish
human
oyster
snake
yeast

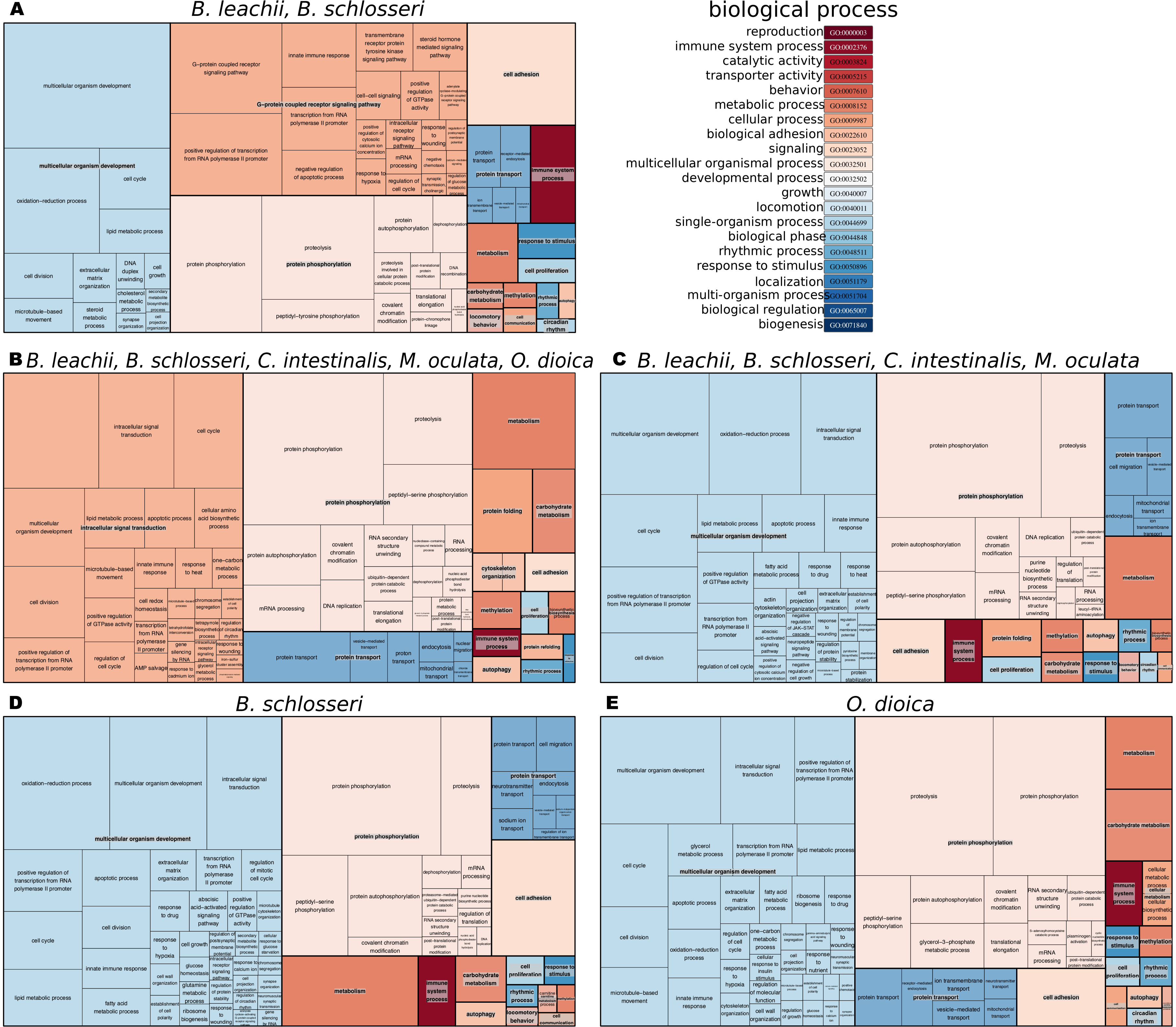
A
B. leachii, B. schlosseri
multicellular organism development
oxidation−reduction process
cell cycle
lipid metabolic process
cell division
extracellular matrix organization
DNA duplex unwinding
cell growth
cholesterol metabolic process
microtubule−based movement
steroid metabolic process
synapse organization
G−protein coupled receptor signaling pathway
innate immune response
transmembrane receptor protein tyrosine kinase signaling pathway
steroid hormone mediated signaling pathway
transcription from RNA polymerase II promoter
cell−cell signaling
positive regulation of GTPase activity
positive regulation of transcription from RNA polymerase II promoter
negative regulation of apoptotic process
mRNA processing
response to hypoxia
regulation of cell cycle
protein phosphorylation
proteolysis
protein autophosphorylation
dephosphorylation
peptidyl−tyrosine phosphorylation
covalent chromatin modification
translational elongation
DNA recombination
cell adhesion
protein transport
immune system process
response to stimulus
metabolism
cell proliferation
carbohydrate metabolism
methylation
rhythmic process
autophagy
locomotory behavior
cell communication
circadian rhythm
biological process
reproduction GO:0000003
immune system process GO:0002376
catalytic activity GO:0003824
transporter activity GO:0005215
behavior GO:0007610
metabolic process GO:0008152
cellular process GO:0009987
biological adhesion GO:0022610
signaling GO:0023052
multicellular organismal process GO:0032501
developmental process GO:0032502
growth GO:0040007
locomotion GO:0040011
single-organism process GO:0044699
biological phase GO:0044848
rhythmic process GO:0048511
response to stimulus GO:0050896
localization GO:0051179
multi-organism process GO:0051704
biological regulation GO:0065007
biogenesis GO:0071840
B
B. leachii, B. schlosseri, C. intestinalis, M. oculata, O. dioica
intracellular signal transduction
cell cycle
multicellular organism development
lipid metabolic process
apoptotic process
cellular amino acid biosynthetic process
cell division
positive regulation of transcription from RNA polymerase II promoter
regulation of cell cycle
protein phosphorylation
proteolysis
peptidyl−serine phosphorylation
protein autophosphorylation
mRNA processing
DNA replication
covalent chromatin modification
RNA secondary structure unwinding
translational elongation
metabolism
protein folding
carbohydrate metabolism
cytoskeleton organization
cell adhesion
methylation
immune system process
autophagy
protein transport
endocytosis
rhythmic process
C
B. leachii, B. schlosseri, C. intestinalis, M. oculata
multicellular organism development
oxidation−reduction process
intracellular signal transduction
cell cycle
lipid metabolic process
apoptotic process
innate immune response
positive regulation of transcription from RNA polymerase II promoter
transcription from RNA polymerase II promoter
cell division
regulation of cell cycle
protein phosphorylation
proteolysis
protein autophosphorylation
peptidyl−serine phosphorylation
DNA replication
mRNA processing
protein transport
cell migration
metabolism
cell adhesion
immune system process
protein folding
methylation
autophagy
rhythmic process
cell proliferation
carbohydrate metabolism
response to stimulus
D
B. schlosseri
oxidation−reduction process
multicellular organism development
intracellular signal transduction
positive regulation of transcription from RNA polymerase II promoter
apoptotic process
cell cycle
cell division
innate immune response
lipid metabolic process
fatty acid metabolic process
protein phosphorylation
proteolysis
peptidyl−serine phosphorylation
protein autophosphorylation
covalent chromatin modification
mRNA processing
protein transport
cell migration
endocytosis
sodium ion transport
cell adhesion
metabolism
immune system process
carbohydrate metabolism
autophagy
cell proliferation
response to stimulus
rhythmic process
locomotory behavior
cell communication
E
O. dioica
multicellular organism development
intracellular signal transduction
positive regulation of transcription from RNA polymerase II promoter
cell cycle
glycerol metabolic process
transcription from RNA polymerase II promoter
lipid metabolic process
apoptotic process
oxidation−reduction process
cell division
microtubule−based movement
innate immune response
proteolysis
protein phosphorylation
peptidyl−serine phosphorylation
protein autophosphorylation
glycerol−3−phosphate metabolic process
translational elongation
mRNA processing
metabolism
carbohydrate metabolism
immune system process
methylation
cell proliferation
rhythmic process
autophagy
circadian rhythm
protein transport
cell adhesion

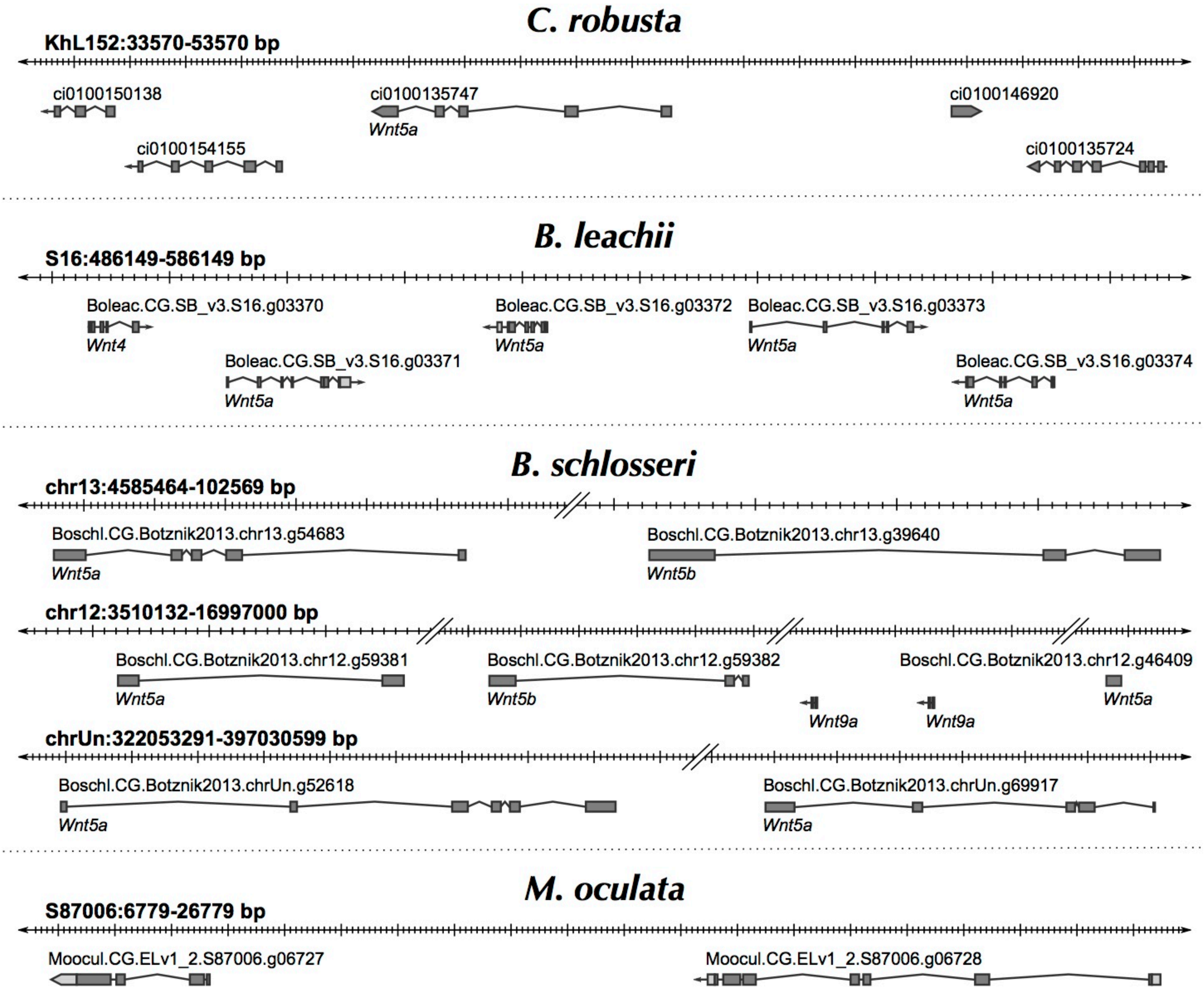
C. robusta
KhL152:33570-53570 bp
ci0100150138
ci0100135747
Wnt5a
ci0100146920
ci0100154155
ci0100135724
B. leachii
S16:486149-586149 bp
Boleac.CG.SB_v3.S16.g03370
Wnt4
Boleac.CG.SB_v3.S16.g03372
Wnt5a
Boleac.CG.SB_v3.S16.g03373
Wnt5a
Boleac.CG.SB_v3.S16.g03371
Wnt5a
Boleac.CG.SB_v3.S16.g03374
Wnt5a
B. schlosseri
chr13:4585464-102569 bp
Boschl.CG.Botznik2013.chr13.g54683
Wnt5a
Boschl.CG.Botznik2013.chr13.g39640
Wnt5b
chr12:3510132-16997000 bp
Boschl.CG.Botznik2013.chr12.g59381
Wnt5a
Boschl.CG.Botznik2013.chr12.g59382
Wnt5b
Wnt9a
Wnt9a
Boschl.CG.Botznik2013.chr12.g46409
Wnt5a
chrUn:322053291-397030599 bp
Boschl.CG.Botznik2013.chrUn.g52618
Wnt5a
Boschl.CG.Botznik2013.chrUn.g69917
Wnt5a
M. oculata
S87006:6779-26779 bp
Moocul.CG.ELv1_2.S87006.g06727
Moocul.CG.ELv1_2.S87006.g06728

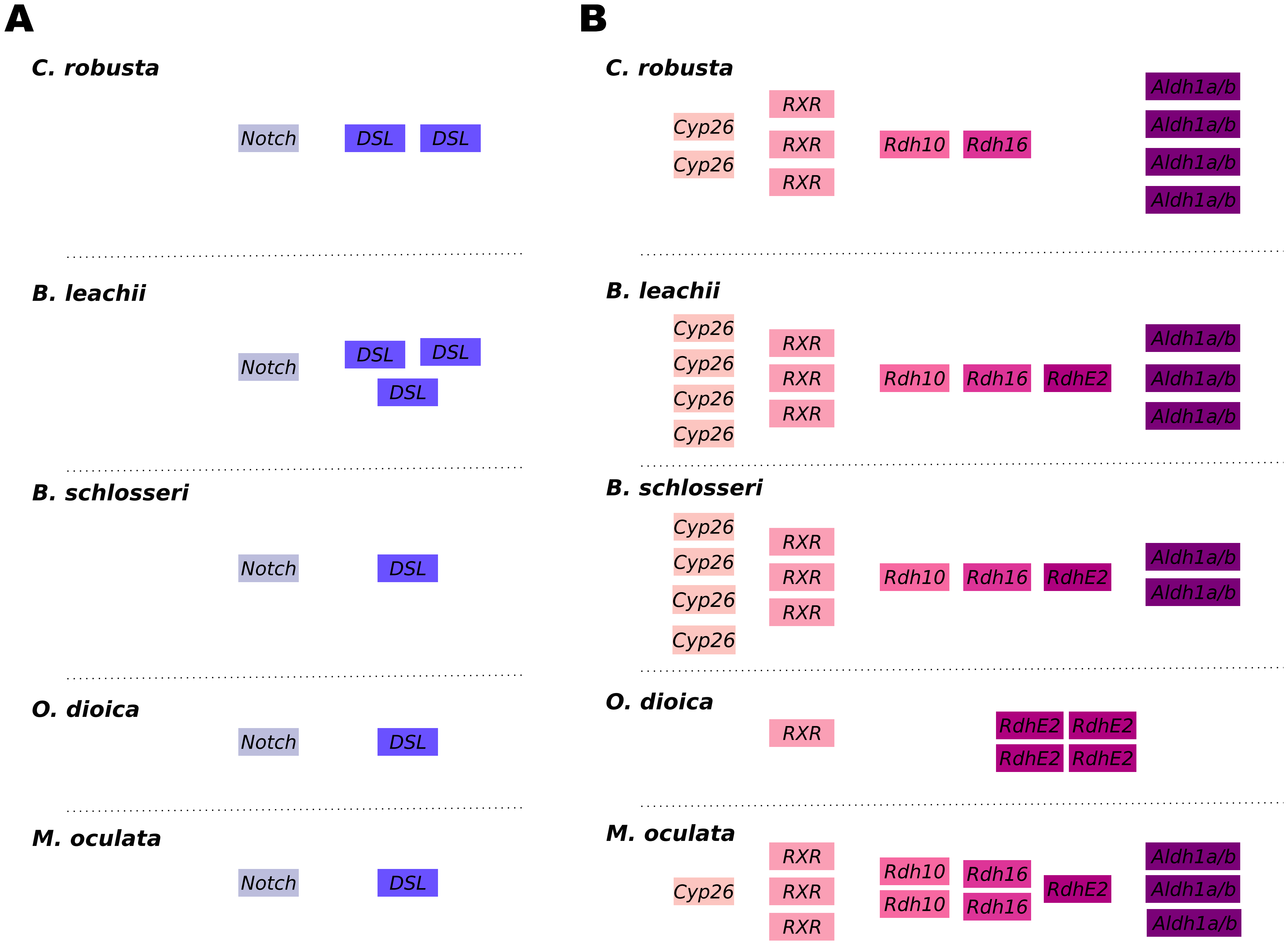
A
B
C. robusta
Notch
DSL
DSL
B. leachii
Notch
DSL
DSL
DSL
B. schlosseri
Notch
DSL
O. dioica
Notch
DSL
M. oculata
Notch
DSL
C. robusta
Cyp26
Cyp26
RXR
RXR
RXR
Rdh10
Rdh16
Aldh1a/b
Aldh1a/b
Aldh1a/b
Aldh1a/b
B. leachii
Cyp26
Cyp26
Cyp26
Cyp26
RXR
RXR
RXR
Rdh10
Rdh16
RdhE2
Aldh1a/b
Aldh1a/b
Aldh1a/b
B. schlosseri
Cyp26
Cyp26
Cyp26
Cyp26
RXR
RXR
RXR
Rdh10
Rdh16
RdhE2
Aldh1a/b
Aldh1a/b
O. dioica
RXR
RdhE2
RdhE2
RdhE2
RdhE2
M. oculata
Cyp26
RXR
RXR
RXR
Rdh10
Rdh10
Rdh16
Rdh16
RdhE2
Aldh1a/b
Aldh1a/b
Aldh1a/b

A Notch pathway

B RA pathway

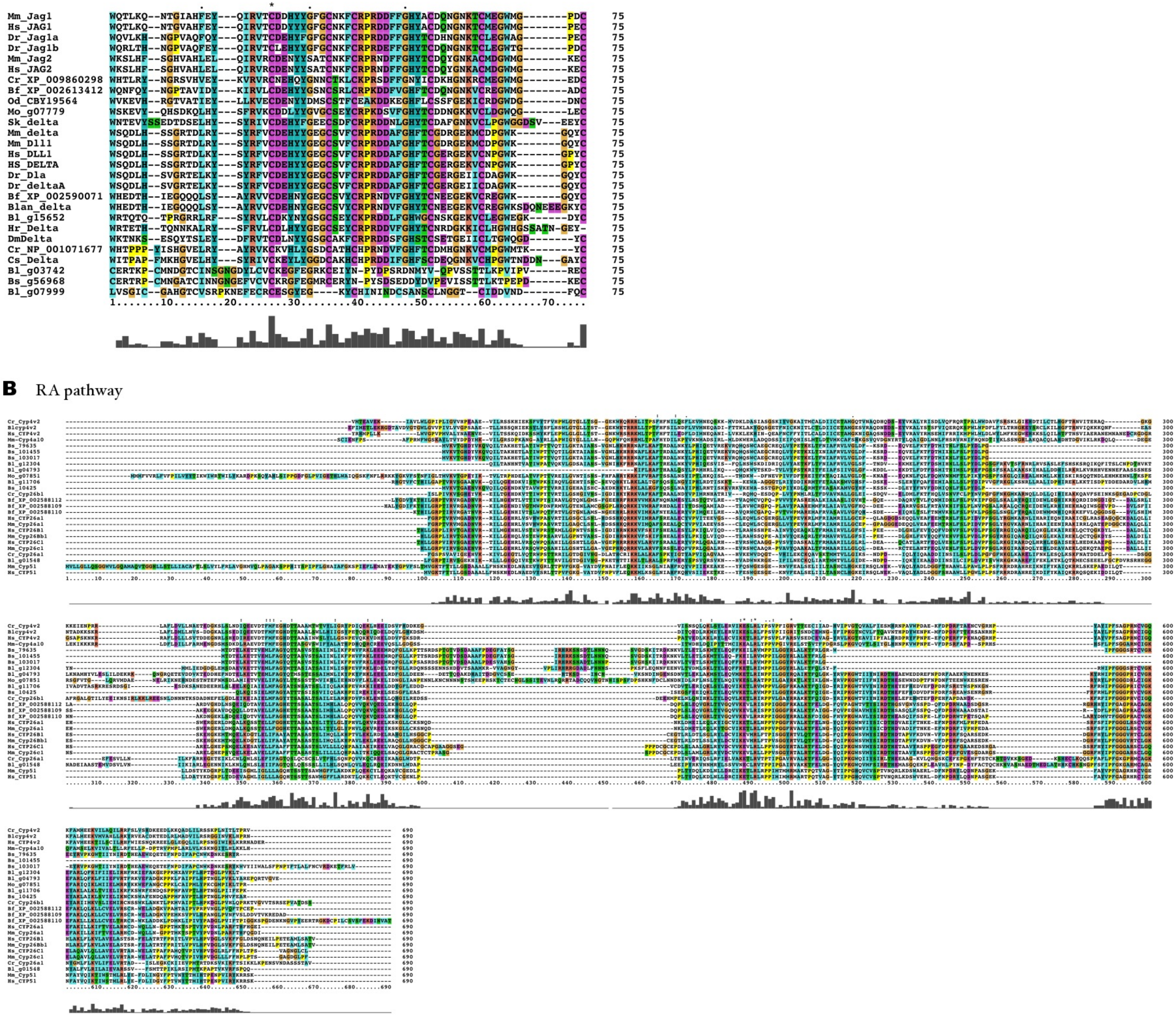

Table S1. Quality metrics of the iterative meta-assembly approach followed for the *de novo* assembly of the *B. leachii* genome. At the end of each round, the assemblies were ranked (see values in brackets), and these assemblies were then combined following the order of their rank. Note that the BUSCO and Glimmer scores were obtained using the default species model, not using the model trained specifically for *B. leachii*. “Mate-pair size” refers to the average length of the mate-pair library used by Metassembler. Assembly “3 kb” from round 3 (in bold) was chosen as the final draft genome.

Round 1

Assembler	N50 [rank]	BUSCO [rank]	Glimmer (all / >1.5 kb) [rank]
AbySS	864 [4]	473 [3]	36953 / 6085 [2]
ALLPATHS-LG	45148 [1]	603 [1]	25933 / 8686 [3]
MaSuRCA_filtered	3060 [2]	500 [2]	54586 / 7121 [1]
MaSuRCA	974 [3]	78 [6]	21907 / 664 [6]
SOAPdenovo2	250 [6]	161 [5]	35832 / 1332 [5]
Velvet	598 [5]	380 [4]	35021 / 3297 [4]

Round 2

Ranking used	N50	BUSCO	Glimmer (all / >1.5 kb)	STAR [rank]
N50	47795	611	24051 / 8575	20537 [2]
BUSCO	45481	607	25170 / 8702	20618 [1]
Glimmer	11860	617	24375 / 8240	17487 [3]

Round 3

Mate-pair size [chosen]	N50	BUSCO	Glimmer	STAR
**3 kb [X]**	**48085**	**616**	**23618 / 8486**	**20509**
8 kb	48702	612	23534 / 8459	9488
15 kb	46966	611	23662 / 8505	20541

Table S2. Conserved domains used to identify the corresponding proteins in tunicate genomes.

**Conserved protein domain family**	**Accession ID**
Delta serrate ligand protein domain	Pfam01414
Notch LNR (lin-notch repeat)	Pfam00066
Wnt	Pfam00110
DIX (dishevelled)	Pfam00778
Cytochrome p450	Pfam00067
17β-HSDXI-like SDR (dehydrogenase)	Cd05339
NR_LBD_RAR	Cd06937
CRD_TK_ROR_like	Cd07459
Aldh-SF	cl11961
SOX-TCF_HMG box	Cd01388
PTKc_Src	Cd05071
